# *In vitro* activity of cefiderocol against European Enterobacterales, including isolates resistant to meropenem and recentβ-lactam/β-lactamase inhibitor combinations

**DOI:** 10.1128/spectrum.04181-23

**Published:** 2024-06-21

**Authors:** Anne Santerre Henriksen, Fabio Arena, Marie Attwood, Rafael Canton, Sören Gatermann, Thierry Naas, Ian Morrissey, Christopher Longshaw, ARTEMIS Study Investigators, Birgit Willinger

**Affiliations:** 1 Medical Affairs, Shionogi B.V., London, United Kingdom; 2 Department of Clinical and Experimental Medicine, University of Foggia, Foggia, Italy; 3 PK/PD Laboratory, North Bristol NHS Trust, Bristol, United Kingdom; 4 Servicio de Microbiología, Hospital Universitario Ramón y Cajal, Instituto Ramón y Cajal de Investigación Sanitaria (IRYCIS), Madrid, Spain; 5 CIBER de Enfermedades Infecciosas (CIBERINFEC), Instituto de Salud Carlos III, Madrid, Spain; 6 Department for Medical Microbiology, Ruhr University, Bochum, Germany; 7 Department of Bacteriology-Hygiene, Hôpital Bicêtre, AP-HP Paris-Saclay, Le Kremlin-Bicêtre, France; 8 Antimicrobial Focus Ltd., Sawbridgeworth, United Kingdom; Instituto de Higiene, Montevideo, Canelones, Uruguay

**Keywords:** cefiderocol, Enterobacterales, *Klebsiella pneumoniae*, meropenem, ceftazidime-avibactam, ceftolozane-tazobactam, meropenem-vaborbactam, imipenem-relebactam, aztreonam-avibactam, cefepime-taniborbactam, resistance, Europe

## Abstract

Carbapenem-resistant Enterobacterales represent a major health threat and have few approved therapeutic options. Enterobacterales isolates were collected from hospitalized inpatients from 49 sites in six European countries (1 January–31 December 2020) and underwent susceptibility testing to cefiderocol and β-lactam/β-lactamase inhibitor combinations. Meropenem-resistant (MIC >8 mg/L) and cefiderocol-susceptible isolates were analyzed by PCR, and cefiderocol-‍resistant isolates by whole-genome sequencing, to identify resistance mechanisms. Overall, 1,909 isolates (including 970 *Klebsiella* spp., 382 *Escherichia coli*, and 244 *Enterobacter* spp.) were collected, commonly from bloodstream infections (43.6%). Cefiderocol susceptibility was higher than approved β-lactam/β-lactamase inhibitor combinations and largely comparable to cefepime-taniborbactam and aztreonam-avibactam against all Enterobacterales (98.1% vs 78.1%–‍97.4% and 98.7%–99.1%, respectively) and Enterobacterales resistant to meropenem (*n* = 148, including 125 *Klebsiella* spp.; 87.8% vs 0%–71.6% and 93.2%–98.6%, respectively), β-lactam/β-lactamase inhibitor combinations (66.7%–‍92.1% vs 0%–‍88.1% and 66.7%–97.9%, respectively), and to both meropenem and β-‌lactam/β-lactamase inhibitor combinations (61.9%–65.9% vs 0%–‍20.5% and 76.2%–97.7%, respectively). Susceptibilities to approved and developmental β-lactam/β-lactamase inhibitor combinations against cefiderocol-resistant Enterobacterales (*n* = 37) were 10.8%–‍56.8% and 78.4%–94.6%, respectively. Most meropenem-resistant Enterobacterales harbored *Klebsiella pneumoniae* carbapenemase (110/148) genes, although metallo-β-lactamase (35/148) and oxacillinase (OXA) carbapenemase (6/148) genes were less common; cefiderocol susceptibility was retained in β-lactamase producers, other than NDM, AmpC, and non-carbapenemase OXA producers. Most cefiderocol-resistant Enterobacterales had multiple resistance mechanisms, including ≥1 iron uptake-related mutation (37/37), carbapenemase gene (33/37), and *ftsI* mutation (24/37). The susceptibility to cefiderocol was higher than approved β-lac‌tam/β-lactamase inhibitor combinations against European Enterobacterales, including meropenem- and β-lactam/β-lactamase inhibitor combination-resistant isolates.

**IMPORTANCE:**

This study collected a notably large number of Enterobacterales isolates from Europe, including meropenem- and β-lactam/β-lactamase inhibitor combination-resistant isolates against which the *in vitro* activities of cefiderocol and developmental β-lactam/β-lactamase inhibitor combinations were directly compared for the first time. The MIC breakpoint for high-dose meropenem was used to define meropenem resistance, so isolates that would remain meropenem resistant with doses clinically available to patients were included in the data. Susceptibility to cefiderocol, as a single active compound, was high against Enterobacterales and was higher than or comparable to available β-lactam/β-lactamase inhibitor combinations. These results provide insights into the treatment options for infections due to Enterobacterales with resistant phenotypes. Early susceptibility testing of cefiderocol in parallel with β-lactam/β-lactamase inhibitor combinations will allow patients to receive the most appropriate treatment option(s) available in a timely manner. This is particularly important when options are more limited, such as against metallo-β-lactamase-producing Enterobacterales.

## INTRODUCTION

Throughout Europe, the occurrence of antimicrobial resistance is widespread (1). Rates of resistance to carbapenems vary across Enterobacterales species, with countries in Southern Europe generally reporting higher rates than Northern Europe (2). For example, in *Klebsiella pneumoniae*, where the overall carbapenem resistance rate was 12% in Europe in 2022, carbapenem resistance rates were ≤1% in Austria, France, Germany, and the United Kingdom (UK; data from 2021 in England only), 5% in Spain, and 25% in Italy (1, 3). There are very few therapeutic options available for infections caused by carbapenem-resistant (CR) Gram-negative bacteria, including CR Enterobacterales (CREs) (1, 2, 4). As such, the World Health Organization has classified CREs as critical priority pathogens (5).

The production of carbapenemases in Enterobacterales is a frequently observed mechanism of resistance in CREs in Europe (6–8). These most commonly include Class A β-‍lactamases, such as *K. pneumoniae* carbapenemase (KPC), with percentages of up to 80% reported in Italy and Greece (9, 10). Other carbapenemases expressed in CREs include the Class D carbapenemase OXA-48, which is prevalent in other Western European countries, such as Spain or France (11–14), and Class B metallo-β-lactamases (MBLs), such as Verona integron MBL (VIM), New Delhi MBL (NDM), and imipenemase (IMP) (4, 6, 9, 15, 16). These MBLs are more commonly observed in Southern European countries such as Greece (13, 17), although there are growing numbers of MBL-producing isolates elsewhere, including in Germany, following increased rates of transmission from Ukraine (18–22). Concerningly, as there are generally limited treatment options for MBL-producing CREs, this unmet need poses a notable threat (23, 24). In addition, increasing numbers of CREs harbor more than one carbapenemase (7, 9, 10) and can also possess resistance mechanisms leading to alteration of membrane permeability and upregulation of efflux pumps (6, 25, 26), contributing to the challenge of combating infections due to CREs.

Cefiderocol is a unique catechol-siderophore cephalosporin approved in Europe for the treatment of infections due to aerobic Gram-negative organisms in adults with limited treatment options (27). The mechanism of action of cefiderocol is similar to other cephalosporins, primarily interacting with penicillin-binding protein (PBP)3 and other PBPs to inhibit peptidoglycan cell wall biosynthesis (28, 29). Of particular relevance against CREs, cefiderocol has demonstrated stability against a range of carbapenemases, including all four classes of β-lactamases (9, 30). In addition, the catechol functional group of cefiderocol chelates free iron, enabling it to be actively transported into bacterial cells via siderophore iron uptake mechanisms and bypass porin channel modification (29, 31). Cefiderocol activity is also broadly unaffected by the upregulation of efflux pumps (32, 33).

There are a range of treatment options available for infections due to Enterobacterales (34, 35). However, options become limited for CRE, even more so depending on the mechanism of resistance, as reflected in the guidance from the European Society of Clinical Microbiology and Infectious Diseases (ESCMID) and the Infectious Diseases Society of America. Available treatments include cefiderocol and various β-lactam/β-lactamase inhibitor (BLBLI) combinations. Ceftazidime-avibactam is active against OXA-48 producers, as well as isolates producing KPC and Class C β-lactamases, and has demonstrated activity against meropenem-resistant Enterobacterales in multiple *in vitro* studies (9, 36–38). Meropenem-vaborbactam and imipenem-relebactam both act against isolates producing Class A and Class C β-lactamases; however, vaborbactam and relebactam are inactive against MBLs and oxacillinases (OXA) (18, 23, 24, 39, 40). Cefepime-taniborbactam and aztreonam-‍avibactam are currently in clinical development and have demonstrated *in vitro* activity against CREs, including isolates producing MBLs (41–43), but neither combination is currently approved for use in Europe (at time of manuscript acceptance). Susceptibilities of cefiderocol and approved BLBLI combinations have been compared against Enterobacterales, including CR and BLBLI combination-‍resistant isolates, in the longitudinal surveillance studies SENTRY and SIDERO (44, 45). Despite this, there remain limited data comparing these antimicrobials with developmental BLBLI combinations against Enterobacterales in Europe.

The aim of this study was to evaluate the *in vitro* activity of cefiderocol, meropenem, BLBLI combinations (approved and in development), and colistin against clinical Gram-negative isolates collected between 1 January and 31 December 2020 across six countries in Europe. Here, we report the results for Enterobacterales isolates, including *Klebsiella* spp., *Escherichia coli*, *Enterobacter* spp., *Citrobacter* spp., *Proteus* spp., *Serratia* spp., and *Morganella morganii*. The results for *Pseudomonas aeruginosa* and *Acinetobacter* spp. isolates collected in this study are reported elsewhere (46).

## RESULTS

### Epidemiology

Overall, 1,909 Enterobacterales were collected from European hospitals, including 970 (50.8%) *Klebsiella* spp., 382 (20.0%) *E. coli*, 244 (12.8%) *Enterobacter* spp., 88 (4.6%) *Serratia* spp., 73 (3.8%) *Citrobacter* spp., 71 (3.7%) *Proteus* spp., and 49 (2.6%) *M*. *morganii* (Table 1; see Table S1 for isolates by country). Isolates were collected from a range of infection sources, the most common being bloodstream [43.6% (*n* = 832)], respiratory tract [22.6% (*n* = 431)], and skin [16.0% (*n* = 306)] (see Table S2 for all infection sources).

**TABLE 1:** *In vitro* activity of cefiderocol, BLBLI combinations, and other relevant antibiotics against Enterobacterales isolates^*a*^^,*b*^

Isolates (*n*)	FDC		MEM		CZA		C‑T		MVB		I-R		ATM-AVI		FEP-TAN		(CST)	
	MIC_90_ (mg/L)	*S* (%)	MIC_90_ (mg/L)	*S* (%)	MIC_90_ (mg/L)	*S* (%)	MIC_90_ (mg/L)	*S* (%)	MIC_90_ (mg/L)	*S* (%)	MIC_90_ (mg/L)	*S* (%)	MIC_90_ (mg/L)	*S* (%)	MIC_90_ (mg/L)	*S* (%)	MIC_90_ (mg/L)	*S* (%)
All Enterobacterales (1,909)^*c*^	2	98.1	4	92.2	1	95.0	>32	78.1	0.5	97.4	1	94.7	≤1	99.1	0.5	98.7	(>8)	(95.3)
*Klebsiella* spp. (970)	2	98.7	>16	86.9	2	95.7	>32	72.2	1	97.1	1	94.7	≤1	99.1	1	99.0	(≤0.25)	(96.3)
*Escherichia coli* (382)	1	99.2	≤0.25	99.5	≤0.25	96.9	1	93.7	≤0.06	99.5	0.25	98.7	≤1	99.2	≤0.25	99.2	(≤0.25)	(98.7)
*Enterobacter* spp. (244)	2	92.2	4	93.9	>16	84.8	>32	61.1	2	93.9	4	86.5	≤1	99.6	0.5	97.5	(>8)	(86.9)
*Citrobacter* spp. (73)	1	98.6	≤0.25	97.3	1	97.3	16	79.5	≤0.06	97.3	0.25	97.3	≤1	98.6	≤0.25	98.6	(≤0.25)	(98.6)
*Proteus* spp. (71)	0.25	100	≤0.25	100	≤0.25	100	4	88.7	0.12	98.6	2	–	≤1	98.6	≤0.25	98.6	(>8)	–
*Morganella morganii* (49)	0.5	100	≤0.25	100	≤0.25	100	1	98.0	0.12	100	2	–	≤1	98.0	≤0.25	100	(>8)	–
*Serratia* spp. (88)	0.5	98.9	≤0.25	100	0.5	100	2	95.5	0.12	100	1	95.5	≤1	98.9	≤0.25	97.7	(>8)	–

^*a*^ ATM-AVI, aztreonam-avibactam; BLBLI, β-lactam/β-lactamase inhibitor; CST, colistin; C-T, ceftolozane-tazobactam; CZA, ceftazidime-avibactam; ECOFF, epidemiological cut-off; EUCAST, European Committee on Antimicrobial Susceptibility Testing; FDC, cefiderocol; FEP-TAN, cefepime-taniborbactam; I-R, imipenem-relebactam; MEM, meropenem; MVB, meropenem-vaborbactam; and S, susceptibility.

^*b*^ Susceptibility was assessed according to EUCAST breakpoints (including high dosage breakpoints and breakpoints for the agent without inhibitor, where necessary), except colistin, for which ECOFF values were used to define the proportion of isolates that could be considered wild-type strains, i.e., those without phenotypically detectable acquired resistance mechanisms. Data on susceptibility to colistin are shown in parentheses as colistin is not recommended for monotherapy and is not associated with a clinical monotherapy breakpoint (as per EUCAST v.14.0 guidance).

^*c*^ Imipenem-relebactam data exclude Morganellaceae (*n* = 1,778) and colistin data exclude Morganellaceae and *Serratia* spp. (*n* = 1,690).

### Susceptibility profiles of isolates

Susceptibility to cefiderocol using European Committee on Antimicrobial Susceptibility Testing (EUCAST) breakpoints was 98.1% for Enterobacterales overall and ranged from 92.2%–100% by species, including 92.2% for *Enterobacter* spp. and 98.9% for *Serratia* spp. isolates; all *Proteus* spp. (*n* = 71) and *M. morganii* (*n* = 49) isolates were cefiderocol susceptible (Table 1). Susceptibility rates using Clinical and Laboratory Standards Institute (CLSI) breakpoints are also reported (Tables S3 and S4). Overall, Enterobacterales susceptibility to cefiderocol across European countries ranged from 96.9% in Italy to 99.0% in the United Kingdom (Table S5A through E). For all Enterobacterales isolates, susceptibility was >90% to all BLBLI combinations, with the exception of ceftolozane-‍tazobactam (78.1%) (Table 1). For each drug, activity by species was broadly similar to the overall activity against all Enterobacterales, with *Enterobacter ‍*spp. showing the most variability (largest variation was observed with ceftolozane-‍tazobactam: 61.1% against *Enterobacter* spp. vs 78.1% against all Enterobacterales) (Table 1). For colistin, 95.3% of isolates (using epidemiological cut-off values) could be considered wild type, i.e., those without phenotypically detectable acquired resistance mechanisms (Table 1).

### Susceptibility profiles of isolates with antibiotic-resistant phenotypes

**Fig 1:**
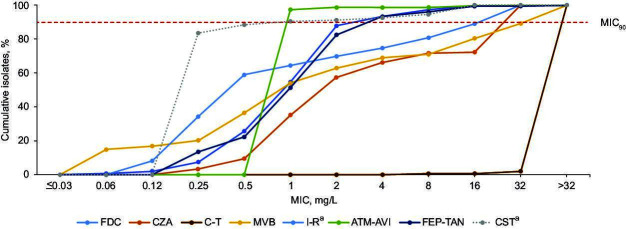
Cumulative MIC distributions of cefiderocol, BLBLI combinations, and other relevant antibiotics against meropenem-resistant Enterobacterales isolates (*n* = 148). Imipenem-relebactam data exclude Morganellaceae, and colistin data exclude Morganellaceae and *Serratia* spp. Data on colistin activity are shown as dashed lines as colistin is not recommended for monotherapy and is not associated with a clinical monotherapy breakpoint (as per EUCAST v.14.0 guidance); ECOFF values were used to define the proportion of isolates that could be considered wild-type strains, i.e., those without phenotypically detectable acquired resistance mechanisms. Resistance to meropenem was defined using a breakpoint of MIC >8 mg/L, relating to high-dose, extended-infusion (2 g, 3-h infusion) meropenem (as per EUCAST v14.0 guidance). ^a^*N* = 146 for imipenem-relebactam and colistin. ATM-AVI, aztreonam-avibactam; BLBLI, β‑lactam/β‑lactamase inhibitor; CST, colistin; C‑T, ceftolozane‑tazobactam; CZA, ceftazidime-avibactam; ECOFF, epidemiological cut-off; EUCAST, European Committee on Antimicrobial Susceptibility Testing; FDC, cefiderocol; FEP-TAN, cefepime-taniborbactam; I-R, imipenem-relebactam; and MVB, meropenem-vaborbactam.

Overall, 7.8% (*n* = 148) Enterobacterales isolates were identified as meropenem resistant (meropenem MIC >8 mg/L), of which 85.1% (*n* = 126) were *Klebsiella* spp. (Table 2; see Fig. 1 for MIC distributions). A high proportion (87.8%) of meropenem-‍resistant Enterobacterales were susceptible to cefiderocol, whereas susceptibility rates were lower for approved BLBLI combinations ceftazidime-‍avibactam (71.6%), meropenem-vaborbactam (70.9%), and imipenem-‍relebactam (77.7%); no meropenem-resistant Enterobacterales were susceptible to ceftolozane-‍tazobactam (Table 2). Developmental BLBLI combinations aztreonam-‍avibactam (98.6%) and cefepime-taniborbactam (93.2%) retained higher activity vs other agents against meropenem-resistant Enterobacterales. These general trends were observed across all countries (Table ‍S6A through E).

**TABLE 2:** *In vitro* activity of cefiderocol, BLBLI combinations, and other relevant antibiotics against Enterobacterales isolates with resistant phenotypes^*a*^^,*b*^

Isolates	*n*	FDC		MEM		CZA		C-T		MVB		I-R		ATM-AVI		FEP-TAN		(CST)	
		MIC_90_ (mg/L)	*S* (%)	MIC_90_ (mg/L)	*S* (%)	MIC_90_ (mg/L)	*S* (%)	MIC_90_ (mg/L)	*S* (%)	MIC_90_ (mg/L)	*S* (%)	MIC_90_ (mg/L)	*S* (%)	MIC_90_ (mg/L)	*S* (%)	MIC_90_ (mg/L)	S (%)	MIC_90_ (mg/L)	S (%)
All Enterobacterales**^*c*^**	1,909	2	98.1	4	92.2	1	95.0	>32	78.1	0.5	97.4	1	94.7	≤1	99.1	0.5	98.7	(>8)	(95.3)
FDC-R	37			>16	51.4	>16	35.1	>32	10.8	>32	56.8	>16	35.1	≤1	94.6	16	78.4	(>8)	(83.3)
MEM-R	148	4	87.8			>16	71.6	>32	0	>32	70.9	>16	69.9	≤1	98.6	4	93.2	(1)	(91.1)
CZA-R	96	8	75.0	>16	56.3			>32	11.5	>32	62.5	>16	31.9	≤1	97.9	8	89.6	(1)	(92.6)
C-T-R	419	2	92.1	>16	64.7	>16	79.7			16	88.1	8	77.7	≤1	96.7	4	95.2	(1)	(92.8)
MVB-R	50	8	68.0	>16	14.0	>16	28.0	>32	0			>16	4.3	>32	86.0	16	68.0	(1)	(91.5)
I-R-R	95	8	74.7	>16	53.7	>16	32.6	>32	4.2	>32	52.6			4	90.5	16	84.2	(1)	(92.3)
FEP-TAN-R	24	8	66.7	>16	58.3	>16	58.3	>32	16.7	>32	33.3	>16	31.8	>32	66.7			(1)	(90.0)
CST-R	79	2	92.4	>16	83.5	2	91.1	>32	63.3	1	94.9	2	91.1	≤1	100	2	97.5		
MEM-R and CZA-R	42	8	61.9					>32	0	>32	16.7	>16	11.9	1	97.6	16	76.2	(8)	(85.7)
MEM-R and MVB-R	43	8	65.1			>16	18.6	>32	0			>16	4.7	1	97.7	16	76.7	(8)	(86.0)
MEM-R and I-R-R	44	8	65.9			>16	20.5	>32	0	>32	11.4			≤1	95.5	16	79.5	(1)	(90.9)
*Klebsiella* spp.	970	2	98.7	>16	86.9	2	95.7	>32	72.2	1	97.1	1	94.7	≤1	99.1	1	99.0	(≤0.25)	(96.3)
MEM-R	127	2	95.3			>16	81.9	>32	0	32	81.9	16	79.5	≤1	98.4	4	97.6	(0.5)	(92.9)
CZA-R	42	4	83.3	>16	45.2			32	7.1	>32	59.5	>16	35.7	≤1	95.2	4	92.9	(1)	(92.9)
C-T-R	270	2	96.3	>16	53.0	>16	85.6			16	89.6	8	81.9	≤1	96.7	2	96.7	(0.5)	(94.4)
MVB-R	28	4	85.7	>16	17.9	>16	39.3	>32	0			>16	7.1	>32	82.1	>32	75.0	(0.5)	(96.4)
I-R-R	51	4	88.2	>16	49.0	>16	47.1	>32	3.9	>32	49.0			32	84.3	8	86.3	(1)	(92.2)
CST-R	36	2	100	>16	75.0	2	91.7	>32	58.3	1	97.2	4	88.9	≤1	100	2	100		
*E. coli*	382	1	99.2	≤0.25	99.5	≤0.25	96.9	1	93.7	≤0.06	99.5	0.25	98.7	≤1	99.2	≤0.25	99.2	(≤0.25)	(98.7)
C-T-R	24	4	87.5	8	91.7	>16	79.2			4	91.7	8	79.2	≤1	91.7	4	95.8	(≤0.25)	(100)
*Enterobacter* spp.	244	2	92.2	4	93.9	>16	84.8	>32	61.1	2	93.9	4	86.5	≤1	99.6	0.5	97.5	(>8)	(86.9)
CZA-R	37	16	59.5	>16	64.9			>32	0	>32	64.9	>16	16.2	≤1	100	8	86.5	(1)	(91.9)
C-T-R	95	8	81.1	>16	84.2	>16	61.1			32	84.2	16	65.3	≤1	98.9	2	93.7	(>8)	(85.3)
I-R-R	33	16	54.5	>16	57.6	>16	6.1	>32	0	>32	54.5			≤1	97.0	16	81.8	(1)	(90.9)
CST-R	32	4	81.3	>16	87.5	1	90.6	>32	56.3	0.5	90.6	2	90.6	≤1	100	2	93.8		

^*a*^ ATM-AVI, aztreonam-avibactam; BLBLI, β-lactam/β-lactamase inhibitor; CST, colistin; C-T, ceftolozane-tazobactam; CZA, ceftazidime-avibactam; ECOFF, epidemiological cut-off; EUCAST, European Committee on Antimicrobial Susceptibility Testing; FDC, cefiderocol; FEP-TAN, cefepime-taniborbactam; I-R, imipenem-relebactam; MEM, meropenem; MVB, meropenem-vaborbactam; R, resistant; and S, susceptibility.

^*b*^ Results are not reported for isolates tested against antibiotics to which they had an expected resistance phenotype. Susceptibility was assessed according to EUCAST breakpoints (including high dosage breakpoints and breakpoints for the agent without inhibitor, where necessary), except colistin, for which ECOFF values were used to define the proportion of isolates that could be considered wild-type strains, i.e., those without phenotypically detectable acquired resistance mechanisms. Data are shown where *n* ≥ 20 isolates were available. Data on susceptibility to colistin are shown in parentheses as colistin is not recommended for monotherapy and is not associated with a clinical monotherapy breakpoint (as per EUCAST v.14.0 guidance).

^*c*^ With the exception of FDC-R isolates, imipenem-relebactam data exclude Morganellaceae (MEM-R, *n* = 146; CZA-R, *n* = 94; C-T-R, *n* = 408; MVB-R, *n* ‍= ‍47; I-R-R, *n* = 95; and FEP-TAN-R, *n* = 22); colistin data exclude Morganellaceae and *Serratia* spp. (FDC-R, *n* = 36; MEM-R, *n* = 146; CZA-R, *n* = 94; C-T-R, *n* = 404; MVB-R, *n* = 47; I-R-R, *n* = 91; and FEP-TAN-R, *n* = 20).

Against Enterobacterales isolates resistant to BLBLI combinations, cefiderocol had higher overall activity than currently approved BLBLI combinations (Table 2). Susceptibility to cefiderocol was highest against ceftolozane-tazobactam-resistant Enterobacterales (92.1%) and ranged from 66.7%–‍75.0% against isolates resistant to ceftazidime-avibactam, meropenem-vaborbactam, imipenem-relebactam, and cefepime-taniborbactam. Against *Klebsiella* spp., *E. coli,* and *Enterobacter* spp. isolates resistant to BLBLI combinations, cefiderocol activity was high, with the exception of ceftazidime-avibactam- and imipenem-relebactam-resistant *Enterobacter* spp. Aztreonam-avibactam retained activity against all BLBLI ‍combination-resistant isolates, with the exception of cefepime-‍taniborbactam-‍resistant isolates, against which there was a similar susceptibility to cefiderocol. Susceptibility to cefepime-taniborbactam in BLBLI ‍combination-‍resistant isolates was lower in comparison with aztreonam-‍avibactam, with percentages similar to cefiderocol. Against Enterobacterales resistant to both meropenem and existing BLBLI combinations, aztreonam-avibactam retained high activity, cefiderocol and cefepime-taniborbactam displayed moderate activity, and other agents were poorly active.

Against cefiderocol-resistant Enterobacterales isolates (*n* = 37), existing BLBLI combinations had low to ‍moderate activity, while both developmental combinations retained moderate to high activity (Table 2). In colistin-resistant Enterobacterales isolates (excluding Morganellaceae and *Serratia* spp.), all agents had good activity. All colistin-resistant *Klebsiella* spp. were susceptible to cefiderocol. Susceptibilities to cefiderocol and BLBLI combinations, with the exception of ceftolozane-tazobactam, were comparable among colistin-resistant *Enterobacter* spp. Susceptibilities for antibiotics against antibiotic-resistant Enterobacterales using CLSI breakpoints are reported in Table S4.

### β-Lactamase genes in meropenem-resistant pathogens

Of the 148 meropenem-resistant Enterobacterales isolates (including 127 *Klebsiella* spp.), 6 were cefiderocol resistant and analyzed by whole-genome sequencing (WGS), while the remaining isolates were analyzed by PCR for the presence of specific β-lactamase genes. Overall, 99.3% (147/148) of isolates harbored at least one carbapenemase gene, including 2.7% (4/148) of isolates harboring two carbapenemase genes (Table ‍S7). The most common β-lactamase genes observed in meropenem-resistant isolates were KPCs [74.3% (110/148); 106 *K. pneumoniae*, 1 *Citrobacter braakii*, 1 *Enterobacter cloacae ‍*complex, 1 *E. coli,* and 1 *Klebsiella* spp.], including *bla*_KPC-3_ (91/110) and *bla*_KPC-2_ (16/110); the majority of isolates harboring KPC genes [90.9% (100/110)] were from Italy (Table 3; Table S7).

**TABLE 3:** Acquired β-lactamase genes in meropenem-resistant Enterobacterales isolates (*n* = 148)^*a*^^,*b*^

Isolate	β-Lactamase group															Negative	Total
	KPC						MBL							OXA			
	Alone	+ESBL	+ESBL + MBL	+ESBL + OXA^*c*^	+MBL	+AmpC	Alone	+ESBL	+ESBL + OXA^*d*^	+OXA^*d*^	+AmpC	+OXA + AmpC^*d*^	+ESBL + AmpC	Alone^*c*^	+ESBL^*e*^		
*Klebsiella pneumoniae*	80	19	2	4	–	1	1	5	1	–	–	1	2	–	4	(1)	121
*Enterobacter bugandensis*	–	–	–	–	–	–	2	–	–	2	–	–	–	1	–	–	5
*Enterobacter cloacae*	–	–	–	–	–	–	2	2	1	–	–	–	–	–	–	–	5
*Enterobacter* spp.	–	–	–	–	–	–	2	–	–	1	–	–	–	–	–	–	3
*E. coli*	–	–	–	1	–	–	–	–	–	–	1	1	–	–	–	–	3
*Klebsiella oxytoca*	–	–	–	–	–	–	1	–	1	–	1	–	–	–	–	–	3
*Enterobacter cloacae* complex	1	–	–	–	–	–	–	–	–	1	–	–	–	–	–	–	2
*Citrobacter braakii*	–	–	–	–	1	–	–	–	–	–	–	–	–	–	–	–	1
*Citrobacter koseri*	–	–	–	–	–	–	1	–	–	–	–	–	–	–	–	–	1
*Klebsiella* spp.	–	1	–	–	–	–	–	–	–	–	–	–	–	–	–	–	1
*Klebsiella variicola*	–	–	–	–	–	–	–	–	1	–	–	–	–	–	–	–	1
*Providencia rettgeri*	–	–	–	–	–	–	1	–	–	–	–	–	–	–	–	–	1
*Providencia stuartii*	–	–	–	–	–	–	–	–	–	–	1	–	–	–	–	–	1
Total	81	20	2	5	1	1	10	7	4	4	3	2	2	1	**4**	(1)	**148**

^*a*^ AmpC, ampicillinase C; ESBL, extended-spectrum β-lactamase; KPC, *Klebsiella pneumoniae* carbapenemase; MBL, metallo-β-lactamase; OXA, oxacillinase; PCR, polymerase chain reaction; VIM, Verona integron-borne metallo-β-lactamase; and WGS, whole genome sequencing.

^*b*^ Data in meropenem-resistant isolates were generated from WGS if isolates were resistant to cefiderocol (*n* = 6) or PCR if isolates were susceptible to cefiderocol (*n* = 142). Isolates are grouped as “negative” if only intrinsic β-lactamase genes were present.

^*c*^ Includes one isolate with *bla*_OXA-48_.

^*d*^ Includes no isolates with *bla*_OXA-48_.

^*e*^ Includes three isolates with *bla*_OXA-48_ and one isolate with *bla*_OXA-232_.

The most commonly observed resistance phenotype was meropenem-resistant Enterobacterales harboring only KPC genes [54.7% (81/148)], followed by isolates co-harboring KPC and ESBL genes [13.5% (20/148)] (Table 3). A total of 23.6% (35/148) of meropenem-resistant Enterobacterales harbored MBL genes, including 27 with NDM [24 *bla*_NDM-1_, 2 *bla*_NDM-4_, and 1 *bla*_NDM-6_; *K. pneumoniae* (10/27) and *Enterobacter* spp. (9/27) were the most common species] and 8 with *bla*_VIM-1_ [most commonly *Enterobacte‍r* spp.‍ (4/8)] (Table S7). A total of 6.8% (10/148) of isolates harbored only MBL genes and 4.7% (7/148) co-harbored MBL and ESBL genes (Table 3). Overall, 4.1% (6/148) of isolates harbored OXA carbapenemase genes (five *bla*_OXA-48_ and one *bla*_OXA-232_), most of which were *K. pneumoniae* (5/6) and co-harbored ESBLs with (1/6) or without (4/6) KPC genes (Table 3; Table S7).

Antimicrobial susceptibilities in meropenem-resistant Enterobacterales according to β-lactamase genes identified are reported in Table 4. All antimicrobials, with the exception of ceftolozane-tazobactam, retained high activity against KPC-producing meropenem-resistant Enterobacterales isolates (*n* = 110). Against MBL producers (*n* = 35), cefiderocol was the only approved antimicrobial that retained moderate–high activity; cefepime-taniborbactam also retained moderate–high activity, and aztreonam-avibactam retained 100% activity.

**TABLE 4:** *In vitro* activity of cefiderocol, BLBLI combinations, and other relevant antibiotics against Enterobacterales isolates resistant to meropenem (MIC >8 mg/L), according to the β-lactamase genes identified^*a*^^,*b*^

Isolates (*n*) producing	FDC			CZA			C-T			MVB			I-R			ATM-AVI			FEP-TAN		
	MIC_90_ (mg/L)	*S* (%)		MIC_90_ (mg/L)	*S* (%)		MIC_90_ (mg/L)	*S* (%)		MIC_90_ (mg/L)	*S* (%)		MIC_90_ (mg/L)	*S* (%)		MIC_90_ (mg/L)	*S* (%)		MIC_90_ (mg/L)	*S* (%)	
		EUCAST	CLSI		EUCAST	CLSI		EUCAST	CLSI		EUCAST	CLSI		EUCAST	CLSI		EUCAST	CLSI		EUCAST	CLSI
OXA (18)	16	44.4	61.1	>16	38.9	38.9	>32	0	0	>32	11.1	11.1	>16	16.7	11.1	<1	94.4	94.4	16	77.8	55.6
Carbapenemases^*c*^ (7)	2	100	100	>16	85.7	85.7	>32	0	0	>32	0	0	16	0	0	32	85.7	85.7	4	100	71.4
Non-carbapenemases^*d*^ (11)	16	9.1	36.4	>16	9.1	9.1	>32	0	0	>32	18.2	18.2	>16	18.2	18.2	<1	100	100	16	63.6	45.5
NDM (27)	16	48.1	70.4	>16	0	0	>32	0	0	>32	0	0	>16	0	0	<1	100	100	16	70.4	44.4
VIM (8)	32	87.5	87.5	>16	0	0	>32	0	0	>32	12.5	0	>16	0	0	<1	100	100	16	75.0	75.0
KPC (110)	2	97.3	99.1	8	91.8	91.8	>32	0	0	4	93.6	91.8	2	91.8	85.5	<1	100	100	2	98.2	91.8
SHV (122)	2	95.9	97.5	>16	82.8	82.8	>32	0	0	32	82.8	80.3	16	80.3	74.6	<1	97.5	97.5	4	96.7	87.7
TEM (71)	2	93.0	97.2	>16	76.1	76.1	>32	0	0	32	76.1	73.2	16	76.1	70.4	<1	98.6	98.6	4	97.2	85.9
CTX-M (37)	8	89.2	89.2	>16	62.2	62.2	>32	0	0	>32	56.8	56.8	>16	59.5	56.8	<1	97.3	97.3	4	91.9	75.7
AmpC^*e*^ (20)	16	55.0	70.0	>16	10.0	10.0	>32	0	0	>32	15.0	10.0	>16	5.0	5.0	<1	95.0	95.0	16	75.0	50.0

^*a*^ AmpC, ampicillinase C; ATM-AVI, aztreonam-avibactam; BLBLI, β-lactam/β-lactamase inhibitor; CLSI, Clinical and Laboratory Standards Institute; C-T, ceftolozane-tazobactam; CZA, ceftazidime-avibactam; EUCAST, European Committee on Antimicrobial Susceptibility Testing; FDC, cefiderocol; FEP-TAN, cefepime-taniborbactam; I, intermediate; KPC, *Klebsiella pneumoniae* carbapenemase; MBL, metallo-β-lactamase; MEM, meropenem; MIC, minimum inhibitory concentration; MVB, meropenem-‍vaborbactam; N/A, not applicable; NDM, New Delhi metallo-β-lactamase; OXA, oxacillinase; S, susceptibility; SHV, sulfhydryl reagent variable; and VIM, Verona integron-borne metallo-β-lactamase.

^*b*^ Resistant phenotypes were defined according to EUCAST breakpoints (v.14.0, 2024). Susceptibility was assessed according to EUCAST (v.14.0, 2024) and CLSI (2023) breakpoints. Of the 148 meropenem-resistant Enterobacterales isolates, 147 harbored at least one acquired β-lactamase gene.

^*c*^ Includes bla_OXA-48_, bla_OXA-181_, and bla_OXA-232_.

^*d*^ Includes bla_OXA-1_, bla_OXA-2_, bla_OXA-9_, bla_OXA-10_, and bla_OXA-101_.

^*e*^ Includes CMY, DHA, ACT, and MIR genes.

### β-Lactamase genes and other potential resistance mechanisms identified in cefiderocol-resistant pathogens

Thirty-seven (1.9%) Enterobacterales isolates were cefiderocol resistant and were analyzed by WGS (Table 5). The most common organism was *E. cloacae* complex [9.8% (10/102) of *E. cloacae* complex isolates], followed by *K. pneumoniae* [1.1% (8/699) of *K. pneumoniae* isolates]. Within each individual species, there was no apparent dominant clone as sequence types (STs) were varied, with the exception of all four *Enterobacter bugandensis* isolates (ST191; from one site in Spain). Of all cefiderocol-resistant Enterobacterales isolates analyzed, 89.2% (33/37) produced at least one carbapenemase gene, mainly metallo-β-lactamase genes [59.5% (22/37); 15 *bla*_NDM-1_ and 7 *bla*_VIM-1_], with 11 such isolates co-producing an OXA-type gene. Only 8.1% (3/37) of isolates harbored KPC genes, all of which were *K. pneumoniae* from Italy. No *bla*_OXA-48_ were identified, but 43.2% (16/37) of isolates produced at least one OXA-type gene (excluding two truncated OXA genes assumed to be non-functional), the most common gene being *bla*_OXA-1_ (14/16). A total of 27.0% (10/37) of cefiderocol-resistant Enterobacterales isolates co-harbored OXA-type and ESBL genes. The non-carbapenemase genes SHV and TEM were identified in 40.5% (15/37) and 43.2% (16/37) of isolates, respectively.

**TABLE 5:** β-Lactamase genes and other potential resistance mechanisms identified in cefiderocol-resistant Enterobacterales isolates (*n* = 37)^*a*^^,*b*^

Iso- late	Country	Organism	FDCMIC (mg/L)	ST	Potential resistance mechanism(s) identified								
					β-lactamase^*c*^	*ftsI*	*ompC*-like	*ompF*-like	*cirA*-like	*fiu*-like	*exbB*	*exbD*	*tonB*
1	Spain	*C. koseri*	8	N/A	CKO-Type; NDM-1	WT	No gross disruption	No gross disruption	N/A	N/A	WT	WT	76–77DEL “EP”; I78V; A134T
2	Spain	*E. bugandensis*	16	191	ACT-52; NDM-1; OXA-1	R2K; I20V; H162R; V563I	No gross disruption	Gene not found	Gene not found	WT	H17Q; I36V; G43S; I49L; V197I; A231V	A81D; D82A; Q90E; A138K	WT
3	Spain	*E. bugandensis*	4	191	ACT-52; NDM-1	R2K; I20V; H162R; V563I	No gross disruption	Gene not found	Gene not found	WT	H17Q; I36V; G43S; I49L; V197I; A231V	A81D; D82A; Q90E; A138K	WT
4	Spain	*E. bugandensis*	16	191	ACT-52; NDM-1; OXA-1	R2K; I20V; H162R; V563I	No gross disruption	Gene not found	Gene not found	WT	H17Q; I36V; G43S; I49L; V197I; A231V	A81D; D82A; Q90E; A138K	WT
5	Spain	*E. bugandensis*	4	191	ACT-52; NDM-1	R2K; I20V; H162R; V563I	No gross disruption	Gene not found	Gene not found	I17V; T32A; I126V; D194E; K244E; E289D; E295D; S401T; N454T; T500L; S565N; M657L; Y658F; Y678K; S684T; K686T	H17Q; I36V; G43S; I49L; V197I; A231V	A81D; D82A; Q90E; A138K	WT
6	Austria	*E. cloacae*	8	78	ACT-24; CTX-M-15; NDM-1; OXA-1; TEM-1	R2K; R152K; H162R; S268A; N277S; T287A; A298S; E378D; V440I; I458V; V563I	No gross disruption	No gross disruption	Gene not found	V19A; T32A; H33Q; Q66H; A90S; I126V; D194E; V203A; R242N; A243K; K244G; E289D; E295D; P298S; N300D; F329Y; A359S; N361S; 77S401A; 7E404Q; N442S; N454T; P457A; T500L; S565N; V571I; L575V; Y658F; G675S; Y678K; K686T; V692L	H17Q; I25V; G43S; I49L; Q60K; M74I; V197I; A231V	I62V; A81D; D82A	A68V
7	France	*E. cloacae*	4	421	ACT-113; CTX-M-15; OXA-1; TEM-1B	R2K; I20V; R152K; H162R; S268A; N277S; T287A; A298S; E378D; V440I; I458V; V563I	No gross disruption	No gross disruption	Gene not found	N142D; V633A	H17Q; G43S; I49L; Q60K; M74I; V197I; A231V	A81D; D82A	S42A; A68V; E117V; R127P; T131A
8	Italy	*E. cloacae*	32	118	ACT-114; CTX-M-9; SHV-12; VIM-1	R2K; R152K; H162R; S268A; N277S; T287A; A298S; E378D; V440I; I458V; V563I	No gross disruption	No gross disruption	Gene not found	Gene not found	H17Q; I25V; G43S; I49L; Q60K; M74I; V197I; A231V	I62V; A81D; D82A	A68V
9	Italy	*E. cloacae*	4	133	ACT-17; SHV-12; TEM-1; VIM-1	R2K; I20V; R152K; H162R; S268A; N277S; T287A; A298S; E378D; V440I; I458V; V563I	Gross disruption	No gross disruption	Gene not found	Gene not found	H17Q; G43S; I49L; Q60K; M74I; V197I; A231V	A81D; D82A	A68V; 77INS "EP"; I78V; T123A; S138N
10	Italy	*E. cloacae*	8	78	ACT-24; NDM-1	R2K; R152K; H162R; S268A; N277S; T287A; A298S; E378D; V440I; I458V; V563I	No gross disruption	Gross disruption	Gene not found	V19A; T32A; H33Q; Q66H; A90S; I126V; D194E; V203A; R242N; A243K; K244G; E289D; E295D; P298S; N300D; F329Y; A359S; N361S; S401A; E404Q; N442S; N454T; P457A; T500L; S565N; V571I; L575V; Y658F; G675S; Y678K; K686T; V692L	H17Q; I25V; G43S; I49L; Q60K; M74I; V197I; A231V	I61V; A81D; D82A	A68V
11	Spain	*E. cloacae*	32	760	CMH-10; CTX-M-9; OXA-1-Trunc^*d*^; SHV-12; VIM-1	H162R	No gross disruption	Gross disruption	A194E; V262L; N478D	WT	WT	WT	WT
12	Spain	*E. cloacae*	4	1,923	CMH-Type	H162R	No gross disruption	No gross disruption	A194E; D231N; V262L	Gene not found	WT	WT	WT
13	Spain	*E. cloacae*	16	456	CMH-9; CTX-M-15	H162R; V440I	No gross disruption	Gene not found	A194E; D231N;V262L; N478D	WT	WT	L7M	WT
14	UK	*E. cloacae*	4	78	ACT-24; NDM-1; TEM-1	R2K; R152K; H162R; S268A; N277S; T287A; A298S; E378D; V440I; I458V; V563I	No gross disruption	No gross disruption	Gene not found	V19A; T32A; H33Q; Q66H; A90S; I126V; D194E; V203A; R242N; A243K; K244G; E289D; E295D; P298S; N300D; F329Y; A359S; N361S; S401A; E404Q; N442S; N454T; P457A; T500L; S565N; V571I; L575V; Y658F; G675S; Y678K; K686T; V692L	H17Q; I25V; G43S; I49L; Q60K; M74I; V197I; A231V	I62V; A81D; D82A	A68V
15	Spain	*E. cloacae complex*	8	191	ACT-52; NDM-1; OXA-1	R2K; I20V; H162R; V563I	No gross disruption	Gene not found	Gene not found	WT	H17Q; I36V; G43S; I49L; V197I; A231V	A81D; D82A; Q90E; A138K	WT
16	Austria	*Enterobacter ludwigii*	16	1,273	ACT-112; NDM-1; OXA-1; OXA-10	H162R; V563I	No gross disruption	Gene not found	Gene not found	Gene not found	I34V; G43S; A46V; I49L; S72L; A80G; V143I; V197I; S204N; A230S; A231V	I61V; A81D; D82Q	WT
17	Italy	*Enterobacter* spp.	4	133	ACT-17; SHV-12; TEM-1; VIM-1	R2K; I20V; R152K; H162R; S268A; N277S; T287A; A298S; E378D; V440I; I458V; V563I	Gross disruption	No gross disruption	Gene not found	Gene not found	H17Q; G43S; I49L; Q60K; M74I; V197I; A231V	A81D; D82A	A68V; 77INS “EP”; I78V; T123A; S138N;
18	Italy	*Enterobacter* spp.	4	133	ACT-17; SHV-12; TEM-1; VIM-1	R2K; I20V; R152K; H162R; S268A; N277S; T287A; A298S; E378D; V440I; I458V; V563I	Gross disruption	No gross disruption	Gene not found	Gene not found	H17Q; G43S; I49L; Q60K; M74I; V197I; A231V	A81D; D82A	A68V; 77INS “EP”; I78V; T123A; S138N
19	Italy	*Enterobacter* spp.	8	418	ACT-115; OXA-1; SHV-12; TEM-1; VIM-1	R2K; I20V; R152K; H162R; S268A; N277S; T287A; A298S; E378D; V440I; I458V; V563I	No gross disruption	No gross disruption	Gene not found	Gene not found	H17Q; G43S; I49L; Q60K; M74I; V197I; A231V	A81D; D82A	A68V; 77INS “EP”; I78V; T123A; S138N
20	Spain	*Enterobacter* spp.	4	90	ACT-56; CTX-M-9; SHV-12	R2K; I20V; R152K; H162R; S268A; N277S; T287A; A298S; E378D; V440I; I458V; V563I	No gross disruption	No gross disruption	Gene not found	Gene not found	H17Q; G43S; I49L; Q60K; M74I; V197I; A231V	A81D; D82A	A68V; 77INS “EP”; I78V; T123A; S138N
21	Austria	*E. coli*	4	127	EC-like; CMY-16; NDM-1; OXA-10; OXA-2	A233T; I332V	No gross disruption	No gross disruption	I174V; D261E;D446E	T38A; S389A; T493A;M513V; V630M; G703D	WT	WT	T51A; 107DEL “Q”; L133P
22	France	*E. coli*	8	354	EC-like; TEM-1; TEM-24	D149E; I332V	No gross disruption	Gross disruption	I174V; N306Y	T38I; S389A; T493A; K508R; M513V; V620G	WT	WT	L28I; T51A; 107DEL “Q”; L133P
23	UK	*E. coli*	4	5,954	CMY-42; EC-like; CTX-M-15; OXA-1; TEM-1	333INS “YRIK”; A413V	No gross disruption	Gross disruption	E465D; E507T; 508–509DEL “TG”; 513–514DEL “RR”; I515V; I547L	V211A; V235I; T367A; G388A; T493A; V495M; M513V	WT	WT	T51A;102INS “KP”; L133P
24	France	*Klebsiella aerogenes*	8	93	Unnamed Class C	WT	No gross disruption	No gross disruption	Gross disruption	N/A	WT	WT	V90A
25	France	*K. aerogenes*	8	634^*e*^	Unnamed Class C; CTX-M-15; OXA-1; TEM-1B	K152R; E461D; V563I	No gross disruption	No gross disruption	D94S; P216S; S620N; S636N	N/A	WT	WT	V90A; R185S; L202V; A225P; K237Q
26	Italy	*K. aerogenes*	>32	92	Unnamed Class C	WT	Gross disruption	No gross disruption	Gross disruption	N/A	WT	WT	V90A
27	Spain	*K. oxytoca*	4	449^*e*^	OXA-101; NDM-1; OXY-1–3; TEM-250	WT	No gross disruption	Gross disruption	E245D	E247D; N279G; G281S; T369I; T372I; V448F	WT	WT	N/A
28	France	*K. pneumoniae*	4	395	SHV-11; CTX-M-15; OXA-1; TEM-1B	WT	No gross disruption	Gross disruption	A134V; N558D	A32T; A287T; S718R	G43R	WT	G63A; 104INS “PKPK”
29	Germany	*K. pneumoniae*	4	76	SHV-1; CTX-M-15	WT	Gross disruption	No gross disruption	A134V; S489F; N558D	D387N; S674N	WT	WT	G63A; P80T; E108K; 108INS “PKPA”; P171A
30	Italy	*K. pneumoniae*	8	3,162	SHV-ESBL; TEM-1B; VIM-1	WT	No gross disruption	No gross disruption	A134V; N558D	N355S; D387N	WT	WT	G63A; 104INS “PKPK”
31	Italy	*K. pneumoniae*	8	1,229	SHV-11; CTX-M-15; NDM-1; OXA-1	WT	Gene not found	No gross disruption	A134V	D387N	WT	WT	G63A; A83V; 104INS “PKPK”
32	Italy	*K. pneumoniae*	4	512	SHV-11; KPC-3; OXA-9-Trunc^*d*^; TEM-1	WT	133INS “GD”	Gross disruption	A134V; N558D	A32T; A287T; S718R	G43R	WT	G63A; P91S; 104INS “PKPK”; P171A; E222Q
33	Italy	*K. pneumoniae*	4	147	SHV-11; CTX-M-15; NDM-1; OXA-1; OXA-9; TEM-1A	WT	No gross disruption	Gross disruption	A134V; N558D	D387N	WT	WT	G63A; A83V; 104INS “PKPK”; P171A; E222Q
34	Italy	*K. pneumoniae*	8	307	CTX-M-15; KPC-31; OXA-1; SHV-28; TEM-1	WT	No gross disruption	No gross disruption	A134V	D387N; S426G	WT	WT	G63A; V68L; A83V; 104INS “PKPK”; P171A
35	Italy	*K. pneumoniae*	4	2,502	SHV-1; KPC-3	WT	No gross disruption	Gross disruption	A134V	D387N	WT	WT	G63A; A83V; 104INS “PKPK”; P171A
36	Austria	*K. variicola*	4	641	LEN-16; NDM-1; OXA-1; OXA-10	WT	No gross disruption	No gross disruption	WT	T31I; A50G; N208S; R227S; N293S; N580D	WT	WT	WT
37	UK	*Serratia marcescens*	4	N/A	SST-Type	P310S	No gross disruption	No gross disruption	N/A	N/A	A48S; T53A; 68INS “A”; I93V; A124S; R147H; S160G; A161P; M264L	WT	E46D; A66S; V160L; K163R; E169N; I171L; R177Q; N184S; V191I; T198S; L219M; Q225E; L232R; I233T; N235T; M246L

^*a*^ AmpC, ampicillinase C; FDC, cefiderocol; KPC, *Klebsiella pneumoniae* carbapenemase; MBL, metallo-β-lactamase; N/A, not applicable; NDM, New Delhi metallo-β-lactamase; OXA, oxacillinase; ST, sequence type; Trunc, truncated; UK, United Kingdom; VIM, Verona integron-borne metallo-β-lactamase; and WT, wild type.

^*b*^ Data were generated by whole-genome sequencing. A gene was considered to have a gross disruption if the coding sequence carried a nonsense mutation, frameshift, indels of >20 codons, or ablation of the canonical stop or start codons without a replacement immediately adjacent and in-frame. Genes were listed to be not found if a BLAST search with the reference gene yielded no hit with *E*-value <1E-25. *C. koseri* or Morganellaceae isolates for which no multilocus sequence typing scheme was available are indicated by “N/A.” There were insufficient data available to confirm whether a gene in *K. aerogenes* with high identity to CMY/ACT/LAT was the chromosomal AmpC of *K. aerogenes*; these genes are indicated as “Unnamed Class C.” Non-β-lactamase genes that were either found to have gross disruptions or mutations or were not found are shown in gray.

^*c*^ A curated summary of the data is shown.

^*d*^ Gene was assumed to be non-functional and was not described as a gene in the results.

^*e*^ Indicates a novel ST.

Most cefiderocol-resistant Enterobacterales [64.9% (24/37)] that were sequenced harbored mutations in all three groups of genes: those related to iron uptake, those encoding porins, and the PBP3-encoding gene *ftsI*. All isolates had mutations in at least one gene related to iron uptake (*cirA*-like, *fiu*-like, *exbB*, *exbD,* or *tonB*); two or more of these genes had mutations or were not detected in 91.9% (34/37) of isolates (Table 5). A high proportion [64.9% (24/37)] also harbored mutations in genes encoding porins and *ftsI*. A total of 18.9% (7/37) of cefiderocol-‍resistant Enterobacterales had *ompC*-like gene mutations or this gene was not detected; 43.2% (16/37) of isolates had *ompF*-like mutations or this gene was not detected.

## DISCUSSION

This study provides additional data on the *in vitro* susceptibilities of cefiderocol and BLBLI combinations, that have been approved or are in development, against a large collection of European Enterobacterales isolates. Data were collected from a greater number of sites per European country compared with the longitudinal surveillance programs SENTRY and SIDERO, which are more geographically spread (44, 45). Susceptibility to cefiderocol was consistently high (>92%) across >1,900 clinical isolates of all Enterobacterales species collected from 49 sites in six countries, in line with previously reported rates in the SENTRY and SIDERO studies (44, 47). BLBLI combinations had variable activity depending on species, but activity was generally high, with the exception of ceftolozane-tazobactam.

Only a small proportion of tested Enterobacterales isolates exhibited resistance to one or more antimicrobials. Overall, cefiderocol and all tested BLBLI combinations consistently demonstrated activity against meropenem-resistant Enterobacterales [other than ceftolozane-tazobactam, which is not a recommended treatment for meropenem-resistant Enterobacterales (34, 35)], which largely comprised *Klebsiella* spp. Of the approved antimicrobials tested, cefiderocol demonstrated the highest activity against meropenem-resistant Enterobacterales (88%), including those with resistance to BLBLI combinations (61.9%–65.9%); high activity of cefiderocol against meropenem-resistant Enterobacterales (47, 48), including those also resistant to ceftazidime-avibactam, has been reported previously (9, 49). Other approved antimicrobials (ceftazidime-avibactam, ceftolozane-tazobactam, and meropenem-vaborbactam) demonstrated variable activity against meropenem-‍resistant Enterobacterales (71.6%, 0%, and 70.9%, respectively). The moderate–‍high activity of ceftazidime-avibactam, which ESCMID guidelines currently recommend for infections caused by carbapenem-resistant Enterobacterales (excluding MBL producers and if active *in vitro*), is in line with previous data (16, 34, 50). However, none of these approved BLBLI combinations demonstrated even moderate activity against meropenem-resistant Enterobacterales also resistant to another BLBLI combination (≤20.5% susceptibility).

The only BLBLI combinations with comparative or higher activity than cefiderocol against meropenem-resistant Enterobacterales isolates were aztreonam-avibactam and cefepime-taniborbactam, which are not yet approved in Europe or anywhere else, although ceftazidime-avibactam plus aztreonam combination therapy appears to be used in the clinic. High activities of these combinations against meropenem-‍resistant Enterobacterales (98.9%) and aztreonam-‍avibactam against meropenem- and ceftazidime-‍avibactam-resistant Enterobacterales (98.9%) have previously been reported (9, 16, 41, 49). It is important to note that if cefiderocol MIC results against meropenem-resistant Enterobacterales were interpreted according to the CLSI susceptibility breakpoint (≤4 mg/L) instead of that recommended by EUCAST (≤2 mg/L) (Table S3), susceptibility to cefiderocol [93.2% (138/148)] would be similar to aztreonam-avibactam (98.6%) and cefepime-taniborbactam (93.2%), based on EUCAST aztreonam and cefepime breakpoints, respectively (Table S4).

Of the 148 meropenem-resistant Enterobacterales isolates (75% from Italy), the majority (74.3%) possessed KPC genes, which is unsurprising due to the high prevalence of KPC-producing CRE in Italy (4, 9, 10). In contrast, MBL [18.2% NDM (16.2% *bla*_NDM-1_); 5.4% VIM (all *bla*_VIM-1_)] and OXA carbapenemase genes [4.1% (3.4% *bla*_OXA-48_)] were less common in our study. With the exception of NDM, AmpC, and non-carbapenemase OXAs, cefiderocol retained good *in vitro* activity when β-lactamase genes were present (Table 4). Cefiderocol has previously been shown to retain activity against carbapenemase-producing Enterobacterales (9, 10). The only agents retaining moderate–high activity against 35 MBL-producing meropenem-‍resistant Enterobacterales in this study were cefiderocol, aztreonam-‍avibactam (100% activity), and cefepime-taniborbactam (Table 4).

Against Enterobacterales isolates resistant to a single BLBLI combination, cefiderocol had moderate to good activity (67%–92%), which was higher compared with ceftazidime-avibactam (28%–80%), meropenem-vaborbactam (33%–88%), and imipenem-relebactam (4%–‍78%). In the SENTRY surveillance study, cefiderocol similarly had moderate activity against BLBLI combination-resistant Enterobacterales isolates (44, 51). Susceptibility to aztreonam-avibactam was high across all resistance phenotypes, with the exception of cefepime-taniborbactam-resistant isolates, where the activity of aztreonam-avibactam was comparable to cefiderocol. Susceptibility to cefepime-taniborbactam was similar to cefiderocol across resistant phenotypes. While cefepime-taniborbactam is a promising alternative for CREs, cefiderocol has proven activity against a wider range of MBLs than taniborbactam-‍containing combinations (49, 52); in the present study, susceptibility to cefiderocol was largely comparable with cefepime-taniborbactam against NDM- and VIM-producing meropenem-resistant isolates when both EUCAST and CLSI breakpoints were considered (Table 4). Previous studies have demonstrated that susceptibility to cefiderocol is retained in Enterobacterales producing IMP and NDM (less so NDM-9), against which taniborbactam-‍containing combinations lack activity (49, 52–54).

Only 1.9% (37/1,909) of Enterobacterales isolates were identified as cefiderocol resistant (the most common species being *E. cloacae* complex). No dominant pattern of STs between pathogens was observed, suggesting cefiderocol resistance is not due to clonal expansion. Of the cefiderocol-resistant (MIC >2 mg/L) isolates, an MIC of 4 mg/L was determined for 51.4% (19/37), all of which would be defined as cefiderocol susceptible according to CLSI breakpoints (MIC ≤4 mg/L). All 18 isolates (<1% of total isolates) that were considered cefiderocol non-susceptible using CLSI criteria (MIC >4 mg/L) harbored β-lactamase genes; *bla*_NDM-1_ or *bla*_VIM-1_ were produced by most (12/18), with few *bla*_KPC_ and no *bla*_OXA-48_ genes. OXA-48-like producers have proven susceptible to cefiderocol, with favorable findings both *in vitro* and *in vivo* (55–58), so this result could be expected. The presence of certain β-lactamases identified in cefiderocol-resistant (MIC >2 mg/L) Enterobacterales in this study, including KPC [8.1% (3/37) of isolates] and non-carbapenemases such as SHV [40.5% (15/37) of isolates], has been suggested to contribute to cefiderocol resistance (59, 60).

With the exception of aztreonam-avibactam and cefepime-taniborbactam, the activity of BLBLI combinations vs cefiderocol-resistant isolates was low; data on BLBLI combinations suggest they do not always demonstrate *in vitro* activity against a wide range of mechanisms of resistance, including certain β-lactamases (37, 39, 40).

All cefiderocol-resistant Enterobacterales isolates had mutations in at least one gene related to iron uptake. Mutations in genes encoding the siderophore receptors CirA and/or Fiu have previously been reported as key mechanisms of cefiderocol resistance in Enterobacterales (*E. cloacae* and *K. pneumoniae*), particularly in NDM-producing isolates (28, 61–69). Specifically, CirA dysfunction has been linked to impaired transport of cefiderocol by iron transport and decreased intracellular concentration in Enterobacterales (61, 62); although interestingly, this has also been attributed to loss of fitness in a cefiderocol-resistant strain of NDM-producing *K. pneumoniae* (63). Mutations in the *cirA*-like gene were commonly observed in cefiderocol-resistant *E. coli* (3/3) and *K. pneumoniae* (8/8), although small proportions (1/3 and 2/8, respectively) were NDM producing. In addition, many cefiderocol-resistant Enterobacterales isolates harbored mutations in the *ftsI* gene for PBP3, although all *K. pneumoniae* isolates had wild-type *ftsI* genes. The relevant contribution of each of these mutations in genes related to iron uptake or PBP3, either alone or in combination with mutations in β-lactamase genes, is not known, nor is any potential impact on the competitive fitness or virulence of these strains. This study was not designed to investigate the mechanisms of resistance in detail, unlike a surveillance study design. Previous data suggest that individual mechanisms of resistance are not sufficient to confer a clinically relevant level of resistance, but that combinations of mechanisms can reduce cefiderocol susceptibility (59); these are consistent with the present study, where most cefiderocol-‍resistant isolates had multiple mechanisms of resistance. Further research on the effect of different combinations of resistance mechanisms is warranted.

The results from this study provide insights into the suitability of available therapeutic options for infections due to Enterobacterales with resistant phenotypes. Cefiderocol demonstrated high levels of *in vitro* activity against Enterobacterales (including those resistant to meropenem and BLBLI combinations, where approved BLBLI combinations did not demonstrate activity) and isolates producing MBLs, which are the most therapeutically challenging carbapenemases to treat in meropenem-resistant Enterobacterales. Therefore, susceptibility testing of cefiderocol at the same time as existing BLBLI combinations would allow clinicians to appropriately choose from all available treatment options.

Furthermore, while colistin is an available treatment option, EUCAST does not currently recommend colistin as a monotherapy, and there is no clinical monotherapy breakpoint (70); therefore, data on susceptibilities to colistin are not reported in full in our study. Additionally, colistin treatment is often associated with high rates of nephrotoxicity (71, 72) and poor tissue penetration, particularly in the lungs (72, 73), so it is less relevant than other agents for this isolate set, which included a high proportion of isolates from the respiratory tract.

There are several limitations to this study. As Enterobacterales isolates were only collected from six European countries, with up to 40 isolates per participating site, there were low numbers of some Enterobacterales with resistant phenotypes, particularly cefiderocol-resistant isolates; this precluded a clear analysis of potential mechanisms of cefiderocol resistance. Although this may be a result of the rarity of cefiderocol resistance in European Enterobacterales, this methodology was not designed to represent a surveillance study. Different methodologies and screening panels were used to analyze a finite selection of potential mechanisms of resistance in meropenem- and cefiderocol-resistant isolates. Although EUCAST recommends characterizing carbapenemases in meropenem-‍resistant Enterobacterales defined by a meropenem MIC >0.125 mg/L (vs MIC >8 mg/L used in this study) (74), this study characterized clinical isolates according to treatment decisions in the clinic, so potential mechanisms of resistance were only investigated in the set of meropenem-resistant isolates that would have been considered resistant in patients. This study did not characterize the expression levels of genes potentially conferring resistance, mutations in clinically relevant intrinsic β-‍lactamase genes, or potential mechanisms of resistance in meropenem-resistant Enterobacterales which were resistant to BLBLI combinations but susceptible to cefiderocol. Therefore, robust interpretations of the resistance mechanisms and the presence or absence of cross-resistance in isolates could not be made. Only the STs of cefiderocol-resistant isolates were identified, so clonal expansion of isolates with other phenotypes was not determined; however, these non-surveillance data would not have accurately reflected clonal epidemiology in Europe. Finally, it is important to note that *in vitro* data cannot replace clinical studies in patients, and *in vitro* activity may not reflect *in vivo* efficacy of a therapy in clinical practice.

### Conclusions

This study builds on existing literature showing that cefiderocol remains the most broadly active approved β-lactam against a large proportion and range of Enterobacterales pathogens in Europe, including those resistant to meropenem or both meropenem and clinically relevant BLBLI combinations, where treatment options are limited. Cefiderocol should be tested in parallel with BLBLI combinations to ensure patients with infection due to CRE receive early treatment with the most appropriate treatment option.

## MATERIALS AND METHODS

### Clinical isolates

Between 1 January and 31 December 2020, Gram-negative clinical isolates from hospitalized inpatients were collected at 49 sites across Austria, France, Germany, Italy, Spain, and the UK (see Table S8 for details of participating centers). Each site was requested to collect 20 *Klebsiella* spp. and 20 other Enterobacterales [methods and results for *P. aeruginosa* and *Acinetobacter* spp. isolates are reported elsewhere (46)]. Isolates included those from all infection sources, with the exception of the urinary tract. Only one isolate of the same genus and species was allowed per patient. Matrix-assisted laser desorption/ionization-time of flight mass spectrometry was used for species identification at IHMA Europe Sàrl (Monthey, Switzerland).

### Antimicrobial susceptibility testing

Antimicrobial susceptibility testing was performed on all collected isolates at IHMA Europe Sàrl. Isolates were stored at −70°C before testing by broth microdilution for the determination of MICs. Antimicrobials tested were cefiderocol, meropenem, ceftazidime-avibactam, ceftolozane-tazobactam, meropenem-vaborbactam, imipenem-relebactam, aztreonam-avibactam, cefepime-taniborbactam, and colistin (see Table S9 for suppliers of agents).

International Organization for Standardization 20776-1 susceptibility testing standards and EUCAST guidance were followed for the preparation of antimicrobials for testing, with the exception of ceftazidime-avibactam, and MIC determinations (75, 76); tryptic soy agar plates containing 5% sheep blood were sourced from Liofilchem (Roseto degli Abruzzi, Italy; product code: 11037), cation-adjusted Mueller-Hinton broth (CAMHB) was sourced from Becton Dickinson (Franklin Lakes, NJ, USA; product code: 212322), and iron-depleted CAMHB (used for cefiderocol testing) was prepared by IHMA Europe Sàrl. Sensititre freeze-dried panels (Thermo Fisher Scientific Inc., Waltham, MA, USA) were used in the preparation of ceftazidime-avibactam for testing, as MIC values were only available for this agent when this validated commercial method was used. All antibiotics were tested daily using the quality control strains *K. ‍pneumoniae* ATCC 700603, *K. pneumoniae* ATCC BAA-2814, and *E. coli* ATCC ‍25922 (recommended by EUCAST) (77). The MIC values for each tested antibiotic were manually read as the lowest concentration inhibiting visible growth. For cefiderocol and meropenem, MIC values were determined more than once; a third MIC determination was carried out if MIC values differed by >1 dilution, when the geometric mean was reported.

### Analysis

Antimicrobial susceptibility results were interpreted in accordance with EUCAST clinical breakpoints (v.14.0, 2024) (78) (see Table S3A). Analyses in accordance with CLSI breakpoints (2023) (79) were also conducted and are presented in the supplementary material. Meropenem resistance was defined using a breakpoint of MIC >8 mg/L, relating to high-dose, extended-infusion (2 g, 3-h infusion) meropenem; similarly, isolates with a meropenem MIC >8 mg/L when tested with a fixed vaborbactam concentration of 8 mg/L were considered resistant to meropenem-vaborbactam. Aztreonam-avibactam and cefepime-taniborbactam do not currently have approved EUCAST MIC breakpoints; breakpoints for aztreonam and cefepime alone were used (Table S3A) (78).

### Identification of β-lactamase genes in meropenem-resistant isolates

Isolates with a meropenem MIC >8 mg/L and a cefiderocol MIC ≤2 mg/L were analyzed by PCR (performed by IHMA Europe Sàrl) to identify the presence of β-lactamase genes that may confer meropenem resistance (see Table S10 for genes and primers used). Data on β-lactamase genes in isolates that were meropenem resistant (MIC >8 mg/L) and cefiderocol resistant (MIC >2 mg/L) were generated by WGS (see below).

DNA extraction was performed from a single colony obtained from a fresh tryptic soy blood agar culture for each isolate, using the QIAGEN TissueLyser II instrument (Hilden, Germany) as per the manufacturer’s instructions. All isolates then underwent PCR amplification and sequencing to screen for the presence of genes encoding clinically relevant β-lactamases: extended-spectrum β-lactamases (ESBLs: *bla*_SHV_, *bla*_TEM_, *bla*_CTX-M_, *bla*_VEB_, *bla*_PER_, and *bla*_GES_), AmpCs (*bla*_ACC_, *bla*_CMY I/MOX_, *bla*_CMY II_, *bla*_DHA_, *bla*_FOX_, and *bla*_ACT-MIR_), and carbapenemases (*bla*_KPC_, *bla*_OXA_, *bla*_NDM_, *bla*_IMP_, *bla*_VIM_, *bla*_SPM_, *bla*_GIM_*,* and *bla*_GES_). Amplicons were sequenced by Fasteris (Geneva, Switzerland) and then analyzed using SeqScape Software 3 (Thermo Fisher Scientific Inc.). Limited sequencing was used to screen *bla*_TEM_ and *bla*_SHV_ to identify TEM-type and SHV-type enzymes containing amino acid substitutions common to ESBLs (*bla*_TEM_: amino acids 104, 164, 238, and 240; *bla*_SHV_: amino acids 146, 179, 238, and 240), and to screen *bla*_CTX-M_ (groups 1, 2, 8, 9, and 25) to identify CTX-M-type enzymes containing the D240G amino acid substitution associated with elevated ceftazidime MICs. Genes encoding SHV-type and TEM-type enzymes were reported as ESBL or original-spectrum β-lactamase genes. The 16S ribosomal DNA in all isolates was also amplified by PCR and sequenced for bacterial identification.

### Identification of β-lactamase genes and other potential resistance mechanisms in cefiderocol-resistant isolates

Isolates with a cefiderocol MIC >2 mg/L were analyzed by WGS to identify possible mechanisms of resistance. DNA isolation was performed using the QIAGEN QIAamp DNA Mini kit, and library preparation was performed using the Illumina DNA Prep kit (San Diego, CA, USA) at International Health Management Associates Inc. (Schaumburg, IL, USA). Libraries were then shipped to Azenta (South Plainfield, NJ, USA), where short-read WGS (2 × 150 base pairs; paired-end) was performed on an Illumina HiSeq platform to a 100× depth of coverage. Quality control was performed using the CheckM lineage workflow (80–82) to ensure low contamination (≤5%) and completeness of assemblies (≥95%) were achieved. Multilocus sequence typing was used to determine the relatedness of isolates; the Achtmann scheme was used for analyzing *E. coli*.

Genomic assemblies were created using the QIAGEN CLC Genomics workbench (v.21.0.5). In order to identify β-lactamase genes of interest, assemblies were queried using the ResFinder database (83) with coverage and identity thresholds of ≥35% and ≥72%, respectively. Genes identified with <100% identity or coverage were evaluated for a variant by pairwise alignment to a reference sequence using the ResFinder database (83). Variants were defined using the Bacterial Antimicrobial Resistance Reference Gene Database from the National Center for Biotechnology Information (BioProject 313047).

Non-β-lactamase genes of interest included those encoding PBP3 (*ftsI*), porins [*ompC*-like and *ompF*-like (in *K. pneumoniae*: *ompK36* and *ompK35*, respectively)], and those related to iron acquisition (*cirA*-like*, fiu*-like, *exbB*, *exbD,* and *tonB*). Genes were analyzed by pairwise alignment and classified as wild type if they had 100% amino acid sequence identity to the species-specific reference sequence (Table S11). Genes were also screened for gross disruption vs species-specific reference sequences (Table S11) and were considered to have gross disruption if the coding sequence carried a nonsense mutation, frameshift, indels of >20 codons, or ablation of the canonical start or stop codons without a replacement immediately adjacent and in-frame. Genes were not considered disrupted if there were ablated start or stop codons immediately adjacent to intact, in-frame start or stop codons. Genes were listed to be not found if a BLAST search with the reference gene yielded no hit with *E*-value <1E-25. Reference genes for several siderophore uptake genes in *Citrobacter koseri*, *Klebsiella oxytoca,* or *Serratia marcescens* were not available, so these genes were not analyzed.

## Data Availability

Whole-genome sequencing variants were defined using the Bacterial Antimicrobial Resistance Reference Gene Database from the National Center for Biotechnology Information (BioProject number: PRJNA313047). Data are available upon reasonable request from Shionogi B.V.

## SUPPLEMENTAL MATERIAL

Supplemental tables spectrum.04181-23-s0001.docx Tables S1–S11.

ASM does not own the copyrights to Supplemental Material that may be linked to, or accessed through, an article. The authors have granted ASM a non-exclusive, world-wide license to publish the Supplemental Material files. Please contact the corresponding author directly for reuse.
