# Supplementary material for: In vitro activity of cefiderocol against European Enterobacterales, including isolates resistant to meropenem and recentβ-lactam/β-lactamase inhibitor combinations
Source: Microbiol Spectr. 2024 Jun 21;12(8):e04181-23. doi: 10.1128/spectrum.04181-23 (PMC11302063; doi:10.1128/spectrum.04181-23)
Supplement: Supplemental tables — Tables S1–S11. [file spectrum.04181-23-s0001.docx]

## SUPPLEMENTARY MATERIALS

**TABLE S1** Enterobacterales isolates by country

| **Isolates** | **France** | **Germany/Austria** | **Italy** | **Spain** | **United Kingdom** |
| --- | --- | --- | --- | --- | --- |
| All collected isolates, *n* | 769 | 570 | 771 | 757 | 493 |
| **All collected Enterobacterales, *n*** | **430** | **333** | **415** | **443** | **288** |
| *Klebsiella* spp. | 213 (49.5) | 175 (52.6) | 221 (53.2) | 221 (49.9) | 140 (48.6) |
| *K. pneumoniae* | 130 (30.2) | 119 (35.7) | 187 (45.1) | 158 (35.7) | 105 (36.5) |
| *K. oxytoca* | 43 (10.0) | 36 (10.8) | 15 (3.6) | 38 (8.6) | 18 (6.3) |
| *K. aerogenes* | 19 (4.4) | 13 (3.9) | 11 (2.7) | 15 (3.4) | 9 (3.1) |
| *K. variicola* | 20 (4.7) | 7 (2.1) | 5 (1.2) | 10 (2.3) | 7 (2.4) |
| *Klebsiella* (unspeciated) | 1 (0.2) | - | 3 (0.7) | - | 1 (0.3) |
| *Escherichia coli* | 61 (14.2) | 65 (19.5) | 93 (22.4) | 94 (21.2) | 69 (24.0) |
| *Enterobacter* spp. | 41 (9.5) | 40 (12.0) | 57 (13.7) | 68 (15.3) | 38 (13.2) |
| *Enterobacter* (unspeciated) | 17 (4.0) | 23 (6.9) | 24 (5.8) | 36 (8.1) | 17 (5.9) |
| *E. cloacae* complex | 20 (4.7) | 14 (4.2) | 31 (7.5) | 23 (5.2) | 14 (4.9) |
| *E. bugandensis* | 4 (0.9) | 3 (0.9) | 2 (0.5) | 9 (2.0) | 7 (2.4) |
| *Serratia* spp. | 21 (4.9) | 15 (4.5) | 12 (2.9) | 21 (4.7) | 19 (6.6) |
| *S. marcescens* | 13 (3.0) | 10 (3.0) | 10 (2.4) | 17 (3.8) | 16 (5.6) |
| *Serratia* (unspeciated) | 5 (1.2) | 4 (1.2) | 1 (0.2) | 3 (0.7) | 1 (0.3) |
| *S. liquefaciens* | 1 (0.2) | 1 (0.3) | 1 (0.2) | 1 (0.2) | 2 (0.7) |
| *S. odorifera* | 1 (0.2) | - | - | - | - |
| *S. proteamaculans* | 1 (0.2) | - | - | - | - |
| *Citrobacter* spp. | 26 (6.0) | 15 (4.5) | 7 (1.7) | 13 (2.9) | 12 (4.2) |
| *C. freundii* | 11 (2.6) | 7 (2.1) | 1 (0.2) | 5 (1.1) | 6 (2.1) |
| *C. koseri* | 9 (2.1) | 6 (1.8) | 5 (1.2) | 5 (1.1) | 4 (1.4) |
| *C. braakii* | 3 (0.7) | 2 (0.6) | 1 (0.2) | 1 (0.2) | - |
| *C. amalonaticus* | 1 (0.2) | - | - | 1 (0.2) | 1 (0.3) |
| *Citrobacter* (unspeciated) | 1 (0.2) | - | - | 1 (0.2) | 1 (0.3) |
| *C. sedlakii* | 1 (0.2) | - | - | - | - |
| *Proteus* spp. | 28 (6.5) | 15 (4.5) | 12 (2.9) | 11 (2.5) | 5 (1.7) |
| *P. mirabilis* | 23 (5.3) | 14 (4.2) | 12 (2.9) | 10 (2.3) | 2 (0.7) |
| *P. vulgaris* | 3 (0.7) | 1 (0.3) | - | 1 (0.2) | 3 (1.0) |
| *P. hauseri* | 1 (0.2) | - | - | - | - |
| *Proteus* (unspeciated) | 1 (0.2) | - | - | - | - |
| *Morganella morganii* | 22 (5.1) | 2 (0.6) | 11 (2.7) | 9 (2.0) | 5 (1.7) |
| *Providencia rettgeri* | 6 (1.4) | 1 (0.3) | - | - | - |
| *Providencia stuartii* | 1 (0.2) | 1 (0.3) | 2 (0.5) | - | - |
| *Raoultella ornithinolytica* | 4 (0.9) | 1 (0.3) | - | 5 (1.1) | - |
| *Raoultella* (unspeciated) | - | - | - | 1 (0.2) | - |
| *Hafnia alvei* | 3 (0.7) | 1 (0.3) | - | - | - |
| *Salmonella* (non-typed) | 3 (0.7) | 1 (0.3) | - | - | - |
| *Pantoea agglomerans* | - | 1 (0.3) | - | - | - |
| *Yersinia enterocolitica* | 1 (0.2) | - | - | - | - |

Data are shown as *n* (%) unless otherwise indicated. Percentages shown are from the number of collected isolates in each country.

**TABLE S2** Enterobacterales isolates by infection source

| **Isolates (*n*)** | **Infection source, n (%)** | | | | | | | |
| --- | --- | --- | --- | --- | --- | --- | --- | --- |
|  | **Respiratory tract** | **Bloodstream** | **Skin** | **Gastro-intestinal** | **Bodily fluids** | **Feces** | **CNS** | **Unknown** |
| **All collected Enterobacterales (1,909)** | **431 (22.6)** | **832 (43.6)** | **306 (16.0)** | **159 (8.3)** | **101 (5.3)** | **67 (3.5)** | **4 (0.2)** | **9 (0.5)** |
| *Klebsiella* spp. (970) | 230 (23.7) | 446 (46.0) | 137 (14.1) | 73 (7.5) | 43 (4.4) | 33 (3.4) | 3 (0.3) | 5 (0.5) |
| *K. aerogenes* (67) | 26 (43.3) | 20 (29.9) | 13 (19.4) | 4 (6.0) | - | 4 (6.0) | - | - |
| *K. oxytoca* (150) | 46 (30.7) | 53 (35.3) | 19 (12.7) | 13 (8.7) | 14 (9.3) | 5 (3.3) | - | - |
| *K. pneumoniae* (699) | 147 (21.0) | 351 (50.2) | 98 (14.0) | 50 (7.2) | 25 (3.6) | 21 (3.0) | 2 (0.3) | 5 (0.7) |
| *Klebsiella* (unspeciated) (5) | 1 (20.0) | 3 (60.0) | 1 (20.0) | - | - | - | - | - |
| *K. variicola* (49) | 10 (20.4) | 19 (38.8) | 6 (12.2) | 6 (12.2) | 4 (8.2) | 3 (6.1) | 1 (2.0) | - |
| *Escherichia coli* (382) | 56 (14.7) | 203 (53.1) | 55 (14.4) | 35 (9.2) | 18 (4.7) | 13 (3.4) | - | 2 (0.5) |
| *Enterobacter* spp. (244) | 66 (27.0) | 82 (33.6) | 47 (19.3) | 27 (11.1) | 12 (4.9) | 8 (3.3) | 1 (0.4) | 1 (0.4) |
| *E. bugandensis* (25) | 9 (16.4) | 6 (24.0) | 5 (20.0) | 3 (12.0) | 1 (4.0) | 1 (4.0) | - | - |
| *E. cloacae* complex (102) | 24 (23.5) | 39 (38.2) | 20 (19.6) | 10 (83.3) | 5 (4.9) | 3 (2.9) | - | 1 (1.0) |
| *Enterobacter* (unspeciated) (117) | 33 (28.2) | 37 (31.6) | 22 (18.8) | 14 (12.0) | 6 (5.1) | 4 (3.4) | 1 (0.9) | - |
| *Serratia* spp. (88) | 31 (35.2) | 32 (36.4) | 16 (18.2) | 3 (3.4) | 4 (4.5) | 2 (2.3) | - | - |
| *S. liquefaciens* (6) | 1 (16.7) | 4 (66.7) | 1 (16.7) | - | - | - | - | - |
| *S. marcescens* (66) | 25 (37.9) | 24 (36.4) | 11 (16.7) | 2 (3.0) | 2 (3.0) | 2 (3.0) | - | - |
| *S. odorifera* (1) | - | 1 (100) | - | - | - | - | - | - |
| *S. proteamaculans* (1) | - | 1 (100) | - | - | - | - | - | - |
| *Serratia* (unspeciated) (14) | 5 (35.7) | 2 (14.3) | 4 (28.6) | 1 (7.1) | 2 (14.3) | - | - | - |
| *Citrobacter* spp. (73) | 18 (24.7) | 18 (24.7) | 10 (13.7) | 11 (15.1) | 10 (13.7) | 5 (6.8) | - | 1 (1.4) |
| *C. amalonaticus* (3) | - | - | 1 (33.3) | 1 (33.3) | - | - | - | 1 (33.3) |
| *C. braakii* (7) | 1 (14.3) | 2 (28.6) | 1 (14.3) | 1 (14.3) | 1 (14.3) | 1 (14.3) | - | - |
| *C. freundii* (30) | 5 (16.7) | 8 (26.7) | 4 (13.3) | 4 (13.3) | 5 (16.7) | 4 (13.3) | - | - |
| *C. koseri* (29) | 11 (37.9) | 7 (24.1) | 4 (13.8) | 4 (13.8) | 3 (10.3) | - | - | - |
| *C. sedlakii* (1) | - | - | - | - | 1 (100) | - | - | - |
| *Citrobacter* (unspeciated) (3) | 1 (33.3) | 1 (33.3) | - | 1 (33.3) | - | - | - | - |
| *Proteus* spp. (71) | 10 (14.1) | 28 (39.4) | 22 (31.0) | 5 (7.0) | 6 (8.5) | - | - | - |
| *P. hauseri* (1) | - | - | 1 (100) | - | - | - | - | - |
| *P. mirabilis* (61) | 7 (11.5) | 25 (41.0) | 19 (31.1) | 5 (8.2) | 5 (8.2) | - | - | - |
| *Proteus* (unspeciated) (1) | - | - | - | - | 1 (100) | - | - | - |
| *P. vulgaris* (8) | 3 (37.5) | 3 (37.5) | 2 (25.0) | - | - | - | - | - |
| *Morganella morganii* (49) | 10 (20.4) | 17 (34.7) | 15 (30.6) | 2 (4.1) | 5 (10.2) | - | - | - |
| *Providencia rettgeri* (7) | - | 2 (28.6) | 2 (28.6) | - | 2 (28.6) | 1 (14.3) | - | - |
| *Providencia stuartii* (4) | - | 2 (50.0) | 1 (25.0) | 1 (25.0) | - | - | - | - |
| *Raoultella ornithinolytica* (10) | 7 (70.0) | - | - | 1 (10.0) | 1 (10.0) | 1 (10.0) | - | - |
| *Raoultella* (unspeciated) (1) | - | 1 (100) | - | - | - | - | - | - |
| *Hafnia alvei* (4) | 3 (75.0) | - | - | 1 (25.0) | - | - | - | - |
| *Salmonella* (non-typed) (4) | - | 1 (25.0) | - | - | - | 3 (75.0) | - | - |
| *Pantoea agglomerans* (1) | - | - | 1 (100) | - | - | - | - | - |
| *Yersinia enterocolitica* (1) | - | - | - | - | - | 1 (100) | - | - |

Percentages are based on number of collected isolates per species/organism.

CNS, central nervous system.

**TABLE S3** Antimicrobial susceptibility testing MIC breakpoints for Enterobacterales

1. EUCAST breakpoints (v.14.0, 2024)^1^

| **Drug** | **MIC breakpoint (mg/L)** | |
| --- | --- | --- |
|  | **S (≤)** | **R (>)** |
| Cefiderocol | 2 | 2 |
| Meropenem^a^ | 8 | 8 |
| Ceftazidime-avibactam^b^ | 8 | 8 |
| Ceftolozane‑tazobactam^b^ | 2 | 2 |
| Meropenem-vaborbactam^a,b,c^ | 8 | 8 |
| Imipenem-relebactam^b,d^ | 2 | 2 |
| Aztreonam-avibactam^a,b,c^ | 4 | 4 |
| Cefepime-taniborbactam^a,c^ | 4 | 4 |
| (Colistin^d,e^) | (2) | (2) |

1. CLSI breakpoints (2023)^2^

| **Drug** | **MIC breakpoint (mg/L)** | | |
| --- | --- | --- | --- |
|  | **S (≤)** | **I** | **R (≥)** |
| Cefiderocol | 4 | 8 | 16 |
| Meropenem | 1 | 2 | 4 |
| Ceftazidime-avibactam | 8/4 | - | 16/4 |
| Ceftolozane‑tazobactam | 2/4 | 4/4 | 8/4 |
| Meropenem-vaborbactam | 4/8 | 8/8 | 16/8 |
| Imipenem-relebactam | 1/4 | 2/4 | 4/4 |
| Aztreonam-avibactam^f^ | 4 | 8 | 16 |
| Cefepime-taniborbactam^f^ | 2 | - | 16 |
| Colistin | - | ≤2 | 4 |

Breakpoints used were those recommended by EUCAST.^1^

^a^EUCAST high dosage breakpoints.

^b^For susceptibility testing purposes, the concentrations of avibactam, tazobactam and relebactam were fixed at 4 mg/L, and the concentration of vaborbactam was fixed at 8 mg/L.

^c^EUCAST breakpoints for the agent without inhibitor.

^d^Excludes Morganellaceae and *Serratia marcescens.*

^e^Colistin is not recommended for monotherapy and is not associated with a clinical monotherapy breakpoint (as per EUCAST v.14.0 guidance); epidemiological cut-off values were used and are shown in parentheses, to define the proportion of isolates that could be considered wild-type strains, i.e., those without phenotypically detectable acquired resistance mechanisms.^1,3^

^f^CLSI breakpoints for the agent without inhibitor.

CLSI, Clinical and Laboratory Standards Institute; EUCAST, European Committee on Antimicrobial Susceptibility Testing; I, intermediate; R, resistant; S, ‍susceptible.

**TABLE S4** *In vitro* activity of cefiderocol, BLBLI combinations and other relevant antibiotics against Enterobacterales isolates, including those with resistant phenotypes, according to CLSI breakpoints

| **Isolates** | ***n*** | **FDC** | | **MEM** | | **CZA** | | **C‑T** | | **MVB** | | **I-R** | | **ATM-AVI** | | **FEP-TAN** | | **(CST)** | |
| --- | --- | --- | --- | --- | --- | --- | --- | --- | --- | --- | --- | --- | --- | --- | --- | --- | --- | --- | --- |
|  |  | **MIC_90_ (mg/L)** | **S (%)** | **MIC_90_ (mg/L)** | **S (%)** | **MIC_90_ (mg/L)** | **S (%)** | **MIC_90_ (mg/L)** | **S (%)** | **MIC_90_ (mg/L)** | **S (%)** | **MIC_90_ (mg/L)** | **S (%)** | **MIC_90_ (mg/L)** | **S (%)** | **MIC_90_ (mg/L)** | **S (%)** | **MIC_90_ (mg/L)** | **I (%)** |
| **All Enterobacterales^a^** | **1,909** | **2** | **99.1** | **4** | **87.2** | **1** | **95.0** | **>32** | **78.1** | **0.5** | **96.9** | **1** | **92.5** | **≤1** | **99.1** | **0.5** | **97.5** | **(>8)** | **(95.3)** |
| FDC-R | 37 |  |  | >16 | 27.0 | >16 | 35.1 | >32 | 10.8 | >32 | 48.6 | >16 | 35.1 | ≤1 | 94.6 | 16 | 64.9 | (>8) | (83.3) |
| MEM-R | 148 | 4 | 93.2 |  |  | >16 | 71.6 | >32 | 0.0 | >32 | 68.9 | >16 | 63.5 | ≤1 | 98.6 | 4 | 82.4 | (1) | (91.1) |
| CZA-R | 96 | 8 | 85.4 | >16 | 21.9% |  |  | >32 | 11.5 | >32 | 56.3 | >16 | 24.0 | ≤1 | 97.9 | 8 | 79.2 | (1) | (92.6) |
| C‑T-R | 419 | 2 | 96.2 | >16 | 42.7 | >16 | 79.7 |  |  | 16 | 85.9 | 8 | 69.5 | ≤1 | 96.7 | 4 | 90.0 | (1) | (92.8) |
| MVB-R | 50 | 8 | 82.0 | >16 | 0.0 | >16 | 28.0 | >32 | 0.0 |  |  | >16 | 0.0 | >32 | 86.0 | 16 | 46.0 | (1) | (91.5) |
| I-R-R | 95 | 8 | 86.3 | >16 | 7.4 | >16 | 32.6 | >32 | 4.2 | >32 | 44.2 |  |  | 4 | 90.5 | 16 | 70.5 | (1) | (92.3) |
| FEP-TAN-R | 24 | 8 | 79.2 | >16 | 12.5 | >16 | 58.3 | >32 | 16.7 | >32 | 33.3 | >16 | 29.2 | >32 | 66.7 |  |  | (1) | (90.0) |
| CST-R | 79 | 2 | 97.5 | >16 | 78.5 | 2 | 91.1 | >32 | 63.3 | 1 | 93.7 | 2 | 82.3 | ≤1 | 100 | 2 | 93.4 |  |  |
| MEM-R and CZA-R | 42 | 8 | 76.2 |  |  |  |  | >32 | 0.0 | >32 | 14.3 | >16 | 11.9 | 1 | 97.6 | 16 | 59.5 | (8) | (85.7) |
| MEM-R and MVB-R | 43 | 8 | 79.1 |  |  | >16 | 18.6 | >32 | 0.0 |  |  | >16 | 0.0 | 1 | 97.7 | 16 | 51.2 | (8) | (86.0) |
| MEM-R and I-R-R | 44 | 8 | 79.5 |  |  | >16 | 20.5 | >32 | 0.0 | >32 | 6.8 |  |  | ≤1 | 95.5 | 16 | 77.3 | (1) | (90.9) |

**Resistant phenotypes were defined according to EUCAST breakpoints (v.14.0, 2024). Susceptibility was assessed according to CLSI breakpoints (2023).** Colistin data are presented using the CLSI intermediate breakpoint and are shown in parentheses as colistin does not have a CLSI susceptibility breakpoint and is not recommended for monotherapy (as per EUCAST v.14.0 and CLSI 2023 guidance).

^a^With the exception of FDC-R isolates, imipenem-relebactam data excludes Morganellaceae (MEM-R, *n* = 146; CZA-R, *n* = 94; C‑T-R, *n* = 408; MVB-R, *n* ‍= ‍47; I-R-R, *n* = 95; FEP-TAN-R, *n* = 22); colistin data excludes Morganellaceae and *Serratia* spp. (FDC-R, *n* = 36; MEM-R, *n* = 146; CZA-R, *n* = 94; C‑T-R, *n* = 404; MVB-R, *n* = 47; I-R-R, *n* = 91; FEP-TAN-R, *n* = 20).
ATM-AVI, aztreonam-avibactam; BLBLI, β‑lactam/β‑lactamase inhibitor; CLSI, Clinical and Laboratory Standards Institute; CST, colistin; C‑T, ceftolozane‑tazobactam; CZA, ceftazidime-avibactam; ECOFF, epidemiological cut-off; EUCAST, European Committee on Antimicrobial Susceptibility Testing; FDC, cefiderocol; FEP-TAN, cefepime-taniborbactam; I, intermediate; I-R, imipenem-relebactam; MEM, meropenem; MIC, minimum inhibitory concentration; MVB, meropenem-vaborbactam; R, resistant; S, susceptibility.

**TABLE S5** *In vitro* activity of cefiderocol, BLBLI combinations and other relevant antibiotics against Enterobacterales isolates, by country

1. France

| **Isolates (n)** | **FDC** | | **MEM** | | **CZA** | | **C‑T** | | **MVB** | | **I-R** | | **ATM-AVI** | | **FEP-TAN** | | **(CST)** | |
| --- | --- | --- | --- | --- | --- | --- | --- | --- | --- | --- | --- | --- | --- | --- | --- | --- | --- | --- |
|  | **MIC_90_ (mg/L)** | **S (%)** | **MIC_90_ (mg/L)** | **S (%)** | **MIC_90_ (mg/L)** | **S (%)** | **MIC_90_ (mg/L)** | **S (%)** | **MIC_90_ (mg/L)** | **S (%)** | **MIC_90_ (mg/L)** | **S (%)** | **MIC_90_ (mg/L)** | **S (%)** | **MIC_90_ (mg/L)** | **S (%)** | **MIC_90_ (mg/L)** | **S (%)** |
| **All Enterobacterales (430)**^a^ | **1** | **98.8** | **≤0.25** | **99.1** | **0.5** | **98.6** | **8** | **84.7** | **≤0.06** | **98.6** | **0.5** | **98.7** | **≤1** | **99.3** | **0.25** | **98.8** | **(≤0.25)** | **(96.0)** |
| *Klebsiella* spp. (213) | 1 | 98.6 | ≤0.25 | 98.6 | 0.5 | 98.1 | 8 | 84.0 | ≤0.06 | 98.6 | 0.5 | 98.1 | ≤1 | 100 | ≤0.25 | 98.6 | (≤0.25) | (98.1) |
| *K. pneumoniae* (130) | 2 | 99.2 | ≤0.25 | 97.7 | 0.5 | 96.9 | 16 | 81.5 | ≤0.06 | 97.7 | 0.5 | 97.7 | ≤1 | 100 | 0.5 | 97.7 | (≤0.25) | (97.7) |
| *K. oxytoca* (43) | 0.5 | 100 | ≤0.25 | 100 | 1 | 100 | 2 | 90.7 | ≤0.06 | 100 | 0.5 | 100 | ≤1 | 100 | ≤0.25 | 100 | (≤0.25) | (97.7) |
| *K. variicola* (20) | 1 | 100 | ≤0.25 | 100 | 0.5 | 100 | 2 | 100 | ≤0.06 | 100 | 0.25 | 100 | ≤1 | 100 | ≤0.25 | 100 | (≤0.25) | (100) |
| *E. coli* (61) | 0.5 | 98.4 | ≤0.25 | 100 | ≤0.25 | 100 | 2 | 90.2 | ≤0.06 | 100 | 0.25 | 100 | ≤1 | 100 | ≤0.25 | 100 | (≤0.25) | (98.4) |
| *Enterobacter* spp. (41) | 2 | 97.6 | ≤0.25 | 100 | 1 | 100 | 16 | 58.5 | ≤0.06 | 97.6 | 0.25 | 97.6 | ≤1 | 97.6 | 2 | 97.6 | (>8) | (85.4) |
| *Proteus* spp. (28) | 0.25 | 100 | ≤0.25 | 100 | ≤0.25 | 100 | 2 | 92.9 | 0.12 | 96.4 | 4 | - | ≤1 | 96.4 | ≤0.25 | 96.4 | (>8) | - |
| *P. mirabilis* (23) | 0.12 | 100 | ≤0.25 | 100 | ≤0.25 | 100 | 2 | 91.3 | 0.12 | 95.7 | 4 | - | ≤1 | 95.7 | ≤0.25 | 95.7 | (>8) | - |
| *Citrobacter* spp. (26) | 1 | 100 | 1 | 100 | 1 | 100 | 16 | 76.9 | ≤0.06 | 100 | 0.25 | 100 | ≤1 | 100 | 0.5 | 100 | (≤0.25) | (100) |
| *Morganella morganii* (22) | 0.25 | 100 | ≤0.25 | 100 | ≤0.25 | 100 | 1 | 100 | 0.12 | 100 | 2 | - | ≤1 | 95.5 | ≤0.25 | 100 | (>8) | - |
| *Serratia* spp. (21) | 0.25 | 100 | ≤0.25 | 100 | 1 | 100 | 1 | 100 | ≤0.06 | 100 | 0.5 | 100 | ≤1 | 100 | ≤0.25 | 100 | (>8) | - |

1. Germany/Austria

| **Isolates (n)** | **FDC** | | **MEM** | | **CZA** | | **C‑T** | | **MVB** | | **I-R** | | **ATM-AVI** | | **FEP-TAN** | | **(CST)** | |
| --- | --- | --- | --- | --- | --- | --- | --- | --- | --- | --- | --- | --- | --- | --- | --- | --- | --- | --- |
|  | **MIC_90_ (mg/L)** | **S (%)** | **MIC_90_ (mg/L)** | **S (%)** | **MIC_90_ (mg/L)** | **S (%)** | **MIC_90_ (mg/L)** | **S (%)** | **MIC_90_ (mg/L)** | **S (%)** | **MIC_90_ (mg/L)** | **S (%)** | **MIC_90_ (mg/L)** | **S (%)** | **MIC_90_ (mg/L)** | **S (%)** | **MIC_90_ (mg/L)** | **S (%)** |
| **All Enterobacterales (333)**^a^ | **1** | **98.5** | **≤0.25** | **97.6** | **0.5** | **97.9** | **2** | **91.9** | **≤0.06** | **97.9** | **0.5** | **96.8** | **≤1** | **98.5** | **0.25** | **99.4** | **(≤0.25)** | **(95.3)** |
| *Klebsiella* spp. (175) | 1 | 98.9 | ≤0.25 | 97.7 | 0.5 | 98.3 | 2 | 94.3 | ≤0.06 | 98.3 | 0.5 | 96.6 | ≤1 | 98.9 | ≤0.25 | 100 | (≤0.25) | (94.3) |
| *K. pneumoniae* (119) | 1 | 99.2 | ≤0.25 | 97.5 | 0.5 | 98.3 | 2 | 94.1 | ≤0.06 | 98.3 | 0.5 | 96.6 | ≤1 | 99.2 | ≤0.25 | 100 | (0.5) | (92.4) |
| *K. oxytoca* (36) | 0.25 | 100 | ≤0.25 | 100 | ≤0.25 | 100 | 0.5 | 100 | ≤0.06 | 100 | 0.25 | 100 | ≤1 | 100 | ≤0.25 | 100 | (≤0.25) | (97.2) |
| *E. coli* (65) | 1 | 98.5 | ≤0.25 | 98.5 | ≤0.25 | 98.5 | 1 | 93.8 | ≤0.06 | 98.5 | 0.25 | 98.5 | ≤1 | 96.9 | 0.5 | 98.5 | (≤0.25) | (100) |
| *Enterobacter* spp. (40) | 1 | 95.0 | ≤0.25 | 95.0 | 0.5 | 95.0 | 8 | 85.0 | ≤0.06 | 95.0 | 0.25 | 95.0 | ≤1 | 100 | ≤0.25 | 100 | (1) | (92.5) |

1. Italy

| **Isolates (n)** | **FDC** | | **MEM** | | **CZA** | | **C‑T** | | **MVB** | | **I-R** | | **ATM-AVI** | | **FEP-TAN** | | **(CST)** | |
| --- | --- | --- | --- | --- | --- | --- | --- | --- | --- | --- | --- | --- | --- | --- | --- | --- | --- | --- |
|  | **MIC_90_ (mg/L)** | **S (%)** | **MIC_90_ (mg/L)** | **S (%)** | **MIC_90_ (mg/L)** | **S (%)** | **MIC_90_ (mg/L)** | **S (%)** | **MIC_90_ (mg/L)** | **S (%)** | **MIC_90_ (mg/L)** | **S (%)** | **MIC_90_ (mg/L)** | **S (%)** | **MIC_90_ (mg/L)** | **S (%)** | **MIC_90_ (mg/L)** | **S (%)** |
| **All Enterobacterales (415)**^a^ | **2** | **96.9** | **>16** | **73.3** | **>16** | **85.1** | **>32** | **50.6** | **2** | **95.7** | **4** | **89.2** | **≤1** | **99.8** | **2** | **98.6** | **(≤0.25)** | **(95.2)** |
| *Klebsiella* spp. (221) | 2 | 96.8 | >16 | 52.5 | >16 | 87.8 | >32 | 32.1 | 4 | 94.6 | 2 | 91.4 | ≤1 | 99.5 | 2 | 98.6 | (≤0.25) | (95.5) |
| *K. pneumoniae* (187) | 2 | 96.8 | >16 | 44.4 | >16 | 87.2 | >32 | 25.7 | 4 | 93.6 | 2 | 90.9 | ≤1 | 100 | 2 | 98.4 | (≤0.25) | (94.7) |
| *E. coli* (93) | 1 | 100 | ≤0.25 | 98.9 | 16 | 89.2 | 2 | 91.4 | ≤0.06 | 98.9 | 0.25 | 96.8 | ≤1 | 100 | ≤0.25 | 100 | (≤0.25) | (97.8) |
| *Enterobacter* spp. (57) | 4 | 89.5 | 8 | 93.0 | >16 | 57.9 | >32 | 31.6 | 8 | 93.0 | 8 | 66.7 | ≤1 | 100 | 0.5 | 98.2 | (>8) | (89.5) |

1. Spain

| **Isolates (n)** | **FDC** | | **MEM** | | **CZA** | | **C‑T** | | **MVB** | | **I-R** | | **ATM-AVI** | | **FEP-TAN** | | **(CST)** | |
| --- | --- | --- | --- | --- | --- | --- | --- | --- | --- | --- | --- | --- | --- | --- | --- | --- | --- | --- |
|  | **MIC_90_ (mg/L)** | **S (%)** | **MIC_90_ (mg/L)** | **S (%)** | **MIC_90_ (mg/L)** | **S (%)** | **MIC_90_ (mg/L)** | **S (%)** | **MIC_90_ (mg/L)** | **S (%)** | **MIC_90_ (mg/L)** | **S (%)** | **MIC_90_ (mg/L)** | **S (%)** | **MIC_90_ (mg/L)** | **S (%)** | **MIC_90_ (mg/L)** | **S (%)** |
| **All Enterobacterales (443)**^a^ | **1** | **97.5** | **1** | **95.5** | **0.5** | **96.2** | **>32** | **81.5** | **0.5** | **97.3** | **2** | **93.4** | **≤1** | **99.5** | **0.5** | **99.1** | **(0.5)** | **(92.8)** |
| *Klebsiella* spp. (221) | 1 | 99.5 | 2 | 95.0 | 1 | 97.3 | >32 | 76.5 | 1 | 98.2 | 2 | 92.8 | ≤1 | 99.5 | 0.5 | 100 | (≤0.25) | (95.0) |
| *K. pneumoniae* (158) | 2 | 100 | 2 | 94.3 | 1 | 98.7 | >32 | 72.2 | 1 | 98.7 | 2 | 91.8 | ≤1 | 99.4 | 0.5 | 100 | (0.5) | (94.3) |
| *K. oxytoca* (38) | 1 | 97.4 | 0.5 | 94.7 | >16 | 89.5 | >32 | 84.2 | 0.12 | 94.7 | 2 | 92.1 | ≤1 | 100 | 0.5 | 100 | (≤0.25) | (97.4) |
| *E. coli* (94) | 1 | 100 | ≤0.25 | 100 | ≤0.25 | 98.9 | 1 | 98.9 | ≤0.06 | 100 | ≤0.12 | 98.9 | ≤1 | 100 | ≤0.25 | 100 | (≤0.25) | (97.9) |
| *Enterobacter* spp. (68) | 4 | 86.8 | >16 | 88.2 | >16 | 86.8 | >32 | 60.3 | 16 | 89.7 | 8 | 85.3 | ≤1 | 100 | 2 | 94.1 | (>8) | (79.4) |
| *Serratia* spp. (21) | 0.25 | 100 | ≤0.25 | 100 | ≤0.25 | 100 | 1 | 100 | ≤0.06 | 100 | 1 | 100 | ≤1 | 95.2 | ≤0.25 | 100 | (>8) | - |

1. United Kingdom

| **Isolates (n)** | **FDC** | | **MEM** | | **CZA** | | **C‑T** | | **MVB** | | **I-R** | | **ATM-AVI** | | **FEP-TAN** | | **(CST)** | |
| --- | --- | --- | --- | --- | --- | --- | --- | --- | --- | --- | --- | --- | --- | --- | --- | --- | --- | --- |
|  | **MIC_90_ (mg/L)** | **S (%)** | **MIC_90_ (mg/L)** | **S (%)** | **MIC_90_ (mg/L)** | **S (%)** | **MIC_90_ (mg/L)** | **S (%)** | **MIC_90_ (mg/L)** | **S (%)** | **MIC_90_ (mg/L)** | **S (%)** | **MIC_90_ (mg/L)** | **S (%)** | **MIC_90_ (mg/L)** | **S (%)** | **MIC_90_ (mg/L)** | **S (%)** |
| **All Enterobacterales (288)**^a^ | **1** | **99.0** | **0.5** | **98.3** | **0.5** | **98.6** | **8** | **86.5** | **0.12** | **97.6** | **0.5** | **96.4** | **≤1** | **97.9** | **0.25** | **97.6** | **(≤0.25)** | **(98.5)** |
| *Klebsiella* spp. (140) | 1 | 100 | 2 | 97.1 | 0.5 | 98.6 | 32 | 82.9 | 0.5 | 95.7 | 1 | 95.7 | ≤1 | 96.4 | 0.5 | 97.1 | (≤0.25) | (99.3) |
| *K. pneumoniae* (105) | 1 | 100 | 2 | 97.1 | 0.5 | 99 | >32 | 82.9 | 0.25 | 95.2 | 1 | 95.2 | ≤1 | 96.2 | 1 | 96.2 | (≤0.25) | (100) |
| *E. coli* (69) | 1 | 98.6 | ≤0.25 | 100 | 0.5 | 100 | 2 | 92.8 | 0.12 | 100 | 0.25 | 100 | ≤1 | 98.6 | ≤0.25 | 97.1 | (≤0.25) | (100) |
| *Enterobacter* spp. (38) | 1 | 97.4 | ≤0.25 | 97.4 | 1 | 94.7 | 16 | 84.2 | 0.12 | 97.4 | 0.5 | 97.4 | ≤1 | 100 | ≤0.25 | 100 | (1) | (92.1) |

Susceptibility was assessed according EUCAST breakpoints (including high dosage breakpoints, and breakpoints for the agent without inhibitor, where necessary), except colistin, for which ECOFF values were used to define the proportion of isolates that could be considered wild-type strains, i.e., those without phenotypically detectable acquired resistance mechanisms.^1^ Data are shown where n isolates ≥20. Colistin is not recommended for monotherapy and is not associated with a clinical monotherapy breakpoint (as per EUCAST v.14.0 guidance).^3^

^a^Imipenem-relebactam data excludes Morganellaceae (France, *n* = 373; Germany/Austria, *n* = 314; Italy, *n* = 390; Spain, *n* = 423; United Kingdom, *n* = 278); colistin data excludes Morganellaceae and *Serratia* spp. (France, *n* = 352; Germany/Austria, *n* = 299; Italy, *n* = 378; Spain, *n* = 402; United Kingdom, *n* ‍= ‍259).

ATM-AVI, aztreonam-avibactam; BLBLI, β‑lactam/β‑lactamase inhibitor; CST, colistin; C‑T, ceftolozane‑tazobactam; CZA, ceftazidime-avibactam; ECOFF, epidemiological cut-off; EUCAST, European Committee on Antimicrobial Susceptibility Testing; FDC, cefiderocol; FEP-TAN, cefepime-taniborbactam; I-R, imipenem-relebactam; MEM, meropenem; MVB, meropenem-vaborbactam; S, susceptibility.

**TABLE S6** *In vitro* activity of cefiderocol, BLBLI combinations and other relevant antibiotics against Enterobacterales isolates with resistance phenotypes, by country

1. France

| **Isolates^a^** | ***n*** | **Susceptibility, %** | | | | | | | |  |
| --- | --- | --- | --- | --- | --- | --- | --- | --- | --- | --- |
|  |  | **FDC** | **MEM** | **CZA** | **C‑T** | **MVB** | **I-R** | **ATM-AVI** | **FEP-TAN** | **(CST)** |
| **C‑T-R** |  |  |  |  |  |  |  |  |  |  |
| **All Enterobacterales** | **66** | **97.0** | **93.9** | **93.9** |  | **90.9** | **92.1** | **97.0** | **93.9** | **(100)** |
| *Klebsiella* spp. | 34 | 97.1 | 91.2 | 91.2 |  | 91.2 | 88.2 | 100 | 94.1 | (100) |
| *K. pneumoniae* | 24 | 95.8 | 87.5 | 87.5 |  | 87.5 | 87.5 | 100 | 91.7 | (100) |

1. Germany/Austria

| **Isolates^a^** | ***n*** | **Susceptibility, %** | | | | | | | |  |
| --- | --- | --- | --- | --- | --- | --- | --- | --- | --- | --- |
|  |  | **FDC** | **MEM** | **CZA** | **C‑T** | **MVB** | **I-R** | **ATM-AVI** | **FEP-TAN** | **(CST)** |
| **C‑T-R** |  |  |  |  |  |  |  |  |  |  |
| **All Enterobacterales** | **27** | **85.2** | **70.4** | **74.1** |  | **74.1** | **61.5** | **85.2** | **96.3** | **(91.7)** |

1. Italy

| **Isolates^a^** | ***n*** | **Susceptibility, %** | | | | | | | |  |
| --- | --- | --- | --- | --- | --- | --- | --- | --- | --- | --- |
|  |  | **FDC** | **MEM** | **CZA** | **C‑T** | **MVB** | **I-R** | **ATM-AVI** | **FEP-TAN** | **(CST)** |
| **MEM-R** |  |  |  |  |  |  |  |  |  |  |
| **All Enterobacterales** | **111** | **95.5** |  | **82.9** | **0** | **84.7** | **83.8** | **100** | **96.4** | **(91.9)** |
| *Klebsiella* spp. | 105 | 95.7 |  | 83.5 | 0 | 91.4 | 87.8 | 100 | 97.8 | (92.8) |
| *K. pneumoniae* | 104 | 96.2 |  | 87.5 | 0 | 89.4 | 88.5 | 100 | 98.1 | (91.3) |
| **CZA-R** |  |  |  |  |  |  |  |  |  |  |
| **All Enterobacterales** | **62** | **82.3** | **69.4** |  | **11.3** | **75.8** | **38.7** | **98.4** | **93.5** | **(96.8)** |
| *Klebsiella* spp. | 27 | 96.2 | 0 |  | 0 | 89.5 | 88.6 | 100 | 98.1 | (91.4) |
| *K. pneumoniae* | 24 | 83.3 | 45.8 |  | 4.2 | 62.5 | 45.8 | 100 | 91.7 | (91.7) |
| *Enterobacter* spp. | 24 | 75.0 | 83.3 |  | 0 | 83.3 | 20.8 | 100 | 95.8 | (100) |
| **C‑T-R** |  |  |  |  |  |  |  |  |  |  |
| **All Enterobacterales** | **205** | **93.7** | **45.9** | **73.2** |  | **91.2** | **78.9** | **99.5** | **97.6** | **(93.4)** |
| *Klebsiella* spp. | 150 | 95.3 | 30.0 | 82.7 |  | 92.0 | 87.3 | 99.3 | 98.0 | (93.3) |
| *K. pneumoniae* | 139 | 95.7 | 25.2 | 83.5 |  | 91.4 | 87.8 | 100 | 97.8 | (92.8) |
| *Enterobacter spp.* | 39 | 84.6 | 89.7 | 38.5 |  | 89.7 | 51.3 | 100 | 97.4 | (92.3) |
| **I-R-R** |  |  |  |  |  |  |  |  |  |  |
| **All Enterobacterales** | **42** | **76.2** | **57.1** | **9.5** | **0** | **59.5** |  | **97.6** | **90.5** | **(97.6)** |

1. Spain

| **Isolates^a^** | ***n*** | **Susceptibility, %** | | | | | | | |  |
| --- | --- | --- | --- | --- | --- | --- | --- | --- | --- | --- |
|  |  | **FDC** | **MEM** | **CZA** | **C‑T** | **MVB** | **I-R** | **ATM-AVI** | **FEP-TAN** | **(CST)** |
| **MEM-R** |  |  |  |  |  |  |  |  |  |  |
| **All Enterobacterales** | **20** | **60.0** |  | **50.0** | **0** | **40.0** | **30.0** | **95.0** | **80.0** | **(80.0)** |
| **C‑T-R** |  |  |  |  |  |  |  |  |  |  |
| **All Enterobacterales** | **82** | **86.6** | **75.6** | **81.7** |  | **85.4** | **68.3** | **98.8** | **95.1** | **(84.1)** |
| *Klebsiella* spp. | 52 | 98.1 | 78.8 | 90.4 |  | 92.3 | 73.1 | 98.1 | 100 | (92.3) |
| *K. pneumoniae* | 44 | 100 | 79.5 | 97.7 |  | 95.5 | 75.0 | 97.7 | 100 | (93.2) |
| *Enterobacter* spp. | 27 | 66.7 | 70.4 | 66.7 |  | 74.1 | 63.0 | 100 | 85.2 | (66.7) |
| **I-R-R** |  |  |  |  |  |  |  |  |  |  |
| **All Enterobacterales** | **28** | **71.4** | **50.0** | **50.0** | **7.1** | **57.1** |  | **96.4** | **85.7** | **(82.1)** |
| **CST-R** |  |  |  |  |  |  |  |  |  |  |
| **All Enterobacterales** | **29** | **79.3** | **86.2** | **86.2** | **55.2** | **89.7** | **82.8** | **100** | **93.1** |  |

1. United Kingdom

| **Isolates^a^** | ***n*** | **Susceptibility, %** | | | | | | | |  |
| --- | --- | --- | --- | --- | --- | --- | --- | --- | --- | --- |
|  |  | **FDC** | **MEM** | **CZA** | **C‑T** | **MVB** | **I-R** | **ATM-AVI** | **FEP-TAN** | **(CST)** |
| **C‑T-R** |  |  |  |  |  |  |  |  |  |  |
| **All Enterobacterales** | **39** | **92.3** | **87.2** | **89.7** |  | **82.1** | **78.9** | **84.6** | **84.6** | **(97.3)** |
| *Klebsiella* spp. | 24 | 100 | 83.3 | 91.7 |  | 75.0 | 75.0 | 79.2 | 83.3 | (100) |

Susceptibility was assessed according EUCAST breakpoints (including high dosage breakpoints, and breakpoints for the agent without inhibitor, where necessary), except colistin, for which ECOFF values were used to define the proportion of isolates that could be considered wild-type strains, i.e., those without phenotypically detectable acquired resistance mechanisms.^1^ Data are shown where n isolates ≥20. Colistin is not recommended for monotherapy and is not associated with a clinical monotherapy breakpoint (as per EUCAST v.14.0 guidance).^3^

^a^Imipenem-relebactam data excludes Morganellaceae; colistin data excludes Morganellaceae and Serratia spp.

ATM-AVI, aztreonam-avibactam; BLBLI, β‑lactam/β‑lactamase inhibitor; CST, colistin; C‑T, ceftolozane‑tazobactam; CZA, ceftazidime-avibactam; EUCAST, European Committee on Antimicrobial Susceptibility Testing; FDC, cefiderocol; FEP-TAN, cefepime-taniborbactam; I-R, imipenem-relebactam; MEM, meropenem; MVB, meropenem-vaborbactam; R, resistant; S, susceptibility.

**TABLE S7** Minimum inhibitory concentration values and β-lactamase genes identified in meropenem-resistant Enterobacterales isolates

| **Country** | **Organism** | **MIC (mg/L)** | | | | | | | | | **β-Lactamase genes identified** |
| --- | --- | --- | --- | --- | --- | --- | --- | --- | --- | --- | --- |
|  |  | **FDC** | **MEM** | **CZA** | **C‑T** | **MVB** | **I-R** | **ATM-AVI** | **FEP-TAN** | **(CST)** |  |
| Austria^a^ | *K. variicola* | 4 | >16 | >16 | >32 | 32 | 16 | <1 | 2 | (<0.25) | LEN-16; NDM-1; OXA-1; OXA-10 |
| Austria | *K. pneumoniae* | 2 | 16 | 16 | >32 | 4 | 8 | 16 | 1 | (<0.25) | DHA-TYPE; SHV-OSBL |
| Austria | *K. pneumoniae* | 2 | >16 | >16 | >32 | 16 | 16 | 2 | 2 | (<0.25) | CTX-M-1-240G; NDM-1; SHV-‍OSBL; TEM-OSBL |
| Austria | *K. pneumoniae* | 0.5 | 16 | 1 | >32 | 32 | 8 | <1 | 4 | (<0.25) | CTX-M-1-24G; OXA-48; SHV-‍OSBL; TEM-OSBL |
| Austria^b^ | *E. coli* | 4 | >16 | >16 | >32 | 16 | 8 | <1 | 4 | (<0.25) | CMY-16; EC-like; NDM-1; OXA-‍10; OXA-2 |
| Austria^b^ | *Enterobacter* sp. | 16 | >16 | >16 | >32 | 16 | 8 | <1 | 1 | (1) | ACT-112; NDM-1; OXA-1; OXA-‍10 |
| Austria | *P. rettgeri* | 1 | >16 | >16 | >32 | >32 | >16 | <1 | 16 | (>8) | NDM-1 |
| Austria^a^ | *E. cloacae* | 8 | >16 | >16 | >32 | 32 | 16 | <1 | 4 | (<0.25) | ACT-24; CTX-M-15; CTX-M-1-24G; NDM-1; OXA-1; TEM-1; TEM-OSBL |
| France | *K. pneumoniae* | 1 | 16 | <0.25 | >32 | 16 | 2 | <1 | 2 | (<0.25) | CTX-M-1-24G; OXA-232; SHV-‍OSBL; TEM-OSBL |
| France | *K. pneumoniae* | 2 | >16 | >16 | >32 | 32 | >16 | <1 | 8 | (<0.25) | CTX-M-9-24D; NDM-4; SHV-‍OSBL; TEM-OSBL |
| France | *K. pneumoniae* | 2 | >16 | >16 | >32 | >32 | 16 | <1 | 1 | (<0.25) | CMY2-TYPE; NDM-1; OXA-181 |
| France | *P. stuartii* | 0.12 | >16 | >16 | >32 | 16 | >16 | <1 | 2 | (>8) | CMY2-TYPE; NDM-4 |
| Italy | *K. pneumoniae* | 2 | >16 | 1 | >32 | 0.12 | 2 | <1 | 0.5 | (<0.25) | KPC-3; SHV-OSBL |
| Italy | *K. pneumoniae* | 1 | >16 | 2 | >32 | 4 | 0.5 | <1 | 2 | (1) | KPC-3; SHV-OSBL |
| Italy | *K. pneumoniae* | 0.5 | >16 | 4 | >32 | 1 | 0.5 | <1 | 1 | (<0.25) | KPC-3; SHV-OSBL; TEM-OSBL |
| Italy | *K. pneumoniae* | 0.5 | >16 | 4 | >32 | 16 | 2 | <1 | 4 | (<0.25) | KPC-2; SHV-OSBL; TEM-OSBL |
| Italy | *K. pneumoniae* | 0.5 | >16 | 4 | >32 | 2 | 0.5 | <1 | 1 | (<0.25) | KPC-3; SHV-OSBL; TEM-OSBL |
| Italy | *K. pneumoniae* | 0.25 | >16 | >16 | >32 | 2 | 0.5 | <1 | 2 | (<0.25) | KPC-3; SHV-OSBL; TEM-OSBL |
| Italy | *K. pneumoniae* | 0.25 | >16 | 1 | >32 | 4 | 0.5 | <1 | 1 | (<0.25) | KPC-2; SHV-OSBL; TEM-OSBL |
| Italy | *K. pneumoniae* | 0.25 | >16 | 1 | >32 | 1 | 0.25 | <1 | 2 | (<0.25) | KPC-2; SHV-OSBL; TME-OSBL |
| Italy | *K. pneumoniae* | 1 | >16 | 2 | >32 | 2 | 1 | <1 | 2 | (<0.25) | KPC-3; SHV-OSBL |
| Italy | *K. pneumoniae* | 0.25 | >16 | 2 | >32 | 4 | 4 | <1 | 2 | (<0.25) | KPC-2; SHV-OSBL; TME-OSBL |
| Italy^a^ | *K. pneumoniae* | 8 | >16 | >16 | >32 | >32 | >16 | <1 | >32 | (<0.25) | CTX-M-15; CTX-M-1-24G; NDM-1; OXA-1; SHV-11; SHV-OSBL |
| Italy | *K. pneumoniae* | 1 | >16 | 2 | >32 | 0.5 | 0.25 | <1 | 1 | (<0.25) | KPC-3; SHV-OSBL |
| Italy | *K. pneumoniae* | 1 | >16 | 2 | >32 | 1 | 0.25 | <1 | 1 | (<0.25) | KPC-3; SHV-OSBL |
| Italy | *K. pneumoniae* | 1 | >16 | 1 | >32 | 1 | 0.5 | <1 | 1 | (0.5) | KPC-3; SHV-OSBL |
| Italy | *K. pneumoniae* | 1 | >16 | 1 | >32 | 2 | 0.5 | <1 | 1 | (<0.25) | KPC-3; SHV-OSBL |
| Italy | *K. pneumoniae* | 1 | >16 | 2 | >32 | 1 | 0.25 | <1 | 1 | (<0.25) | KPC-3; SHV-OSBL |
| Italy | *K. pneumoniae* | 1 | >16 | 1 | >32 | 1 | 0.25 | <1 | 1 | (<0.25) | KPC-3; SHV-OSBL |
| Italy | *K. pneumoniae* | 2 | >16 | 2 | >32 | 0.5 | 0.25 | <1 | 2 | (<0.25) | KPC-3; SHV-OSBL |
| Italy | *K. pneumoniae* | 0.5 | >16 | 1 | >32 | >0.05 | <0.12 | <1 | <0.25 | (<0.25) | KPC-3; SHV-OSBL |
| Italy | *K. pneumoniae* | 1 | >16 | 1 | >32 | 1 | 0.25 | <1 | 1 | (<0.25) | KPC-3; SHV-OSBL |
| Italy | *K. pneumoniae* | 0.5 | >16 | 1 | >32 | 1 | 0.5 | <1 | 1 | (<0.25) | KPC-3; SHV-OSBL |
| Italy | *K. pneumoniae* | 1 | >16 | 1 | >32 | 1 | 0.25 | <1 | 2 | (<0.25) | KPC-3; SHV-OSBL |
| Italy | *K. pneumoniae* | 0.5 | >16 | 2 | >32 | 4 | 0.5 | <1 | 4 | (0.5) | KPC-3; SHV-OSBL |
| Italy | *K. pneumoniae* | 0.5 | >16 | 2 | >32 | 4 | 0.5 | <1 | 4 | (0.5) | KPC-3; SHV-OSBL |
| Italy | *K. pneumoniae* | 1 | >16 | 1 | >32 | 1 | 0.25 | <1 | 1 | (<0.25) | KPC-3; SHV-OSBL |
| Italy | *K. pneumoniae* | 2 | >16 | 1 | >32 | 2 | 0.25 | <1 | 2 | (<0.25) | KPC-3; SHV-OSBL |
| Italy | *K. pneumoniae* | 2 | >16 | 1 | >32 | 2 | 0.25 | <1 | 2 | (<0.25) | CTX-M-9-24D; KPC-3; SHV-‍OSBL |
| Italy | *K. pneumoniae* | 0.5 | 16 | >16 | >32 | >0.05 | <0.12 | <1 | <0.25 | (<0.25) | CTX-M-1-24G; KPC-3; SHV-‍OSBL; TEM-OSBL |
| Italy | *K. pneumoniae* | 1 | >16 | 2 | >32 | 8 | 2 | <1 | 2 | (<0.25) | KPC-3; SHV-OSBL; TEM-OSBL |
| Italy | *K. pneumoniae* | 0.5 | >16 | 1 | >32 | 1 | 0.25 | <1 | 2 | (>8) | KPC-3; SHV-OSBL |
| Italy | *K. pneumoniae* | 1 | >16 | 2 | >32 | 0.5 | 0.25 | <1 | 2 | (8) | KPC-3; SHV-OSBL |
| Italy | *K. pneumoniae* | 2 | >16 | >16 | >32 | >0.05 | 0.25 | <1 | <0.25 | (<0.25) | CTX-M-1-24G; KPC-3; SHV-‍OSBL; TEM-OSBL |
| Italy | *K. pneumoniae* | 2 | >16 | >16 | >32 | >32 | >16 | <1 | 1 | (4) | CTX-M-1-24G; NDM-1; SHV-‍OSBL; TEM-OSBL |
| Italy | *K. pneumoniae* | 0.5 | >16 | 0.5 | >32 | 0.5 | 0.5 | <1 | 2 | (2) | CTX-M-1-24G; KPC-3; SHV-‍OSBL; TEM-OSBL |
| Italy | *K. pneumoniae* | 2 | >16 | >16 | >32 | >32 | >16 | <1 | 1 | (<0.25) | NDM-6; SHV-OSBL; TEM-OSBL |
| Italy | *K. pneumoniae* | 0.5 | >16 | 1 | >32 | 0.5 | 1 | <1 | 2 | (8) | KPC-2; SHV-OSBL |
| Italy^b^ | *K. pneumoniae* | 2 | 16 | 1 | >32 | 0.12 | 0.5 | <1 | <0.25 | (<0.25) | CTX-M-15; KPC-3; OXA-9; SHV-‍11; TEM-1 |
| Italy | *K. pneumoniae* | 0.5 | >16 | >16 | >32 | >32 | 16 | <1 | 8 | (<0.25) | CTX-M-1-24G; KPC-3; SHV-‍OSBL; TEM-OSBL; VIM-1 |
| Italy | *K. pneumoniae* | 2 | >16 | >16 | >32 | 0.25 | 0.25 | <1 | 1 | (<0.25) | KPC-3; SHV-OSBL; TEM-OSBL |
| Italy | *K. pneumoniae* | 1 | >16 | 0.5 | >32 | 0.5 | <0.12 | <1 | 2 | (<0.25) | KPC-3; SHV-OSBL; TEM-OSBL |
| Italy | *K. pneumoniae* | 0.5 | >16 | 2 | >32 | 0.5 | 0.5 | <1 | 2 | (8) | KPC-3; SHV-OSBL |
| Italy | *K. pneumoniae* | 1 | >16 | 8 | >32 | 0.12 | <0.12 | <1 | <0.25 | (<0.25) | CTX-M-1-24G; KPC-2; SHV-OSBL; TEM-OSBL |
| Italy | *K. pneumoniae* | 0.25 | >16 | 0.5 | >32 | >0.05 | 0.25 | <1 | <0.25 | (<0.25) | CTX-M-1-240G; KPC-2; SHV-OSBL; TEM-OSBL |
| Italy | *K. pneumoniae* | 0.5 | 16 | 1 | >32 | >0.05 | 0.25 | <1 | <0.25 | (<0.25) | CTX-M-1-24G; KPC-2; SHV-OSBL; TEM-OSBL |
| Italy | *K. pneumoniae* | 1 | >16 | 1 | >32 | 0.5 | 0.25 | <1 | 1 | (<0.25) | KPC-3; SHV-OSBL; TEM-OSBL |
| Italy | *K. pneumoniae* | 1 | 16 | <0.25 | >32 | <0.06 | <0.12 | <1 | <0.25 | (<0.25) | CTX-M-1-240G; KPC-2; SHV-OSBL; TEM-OSBL |
| Italy | *K. pneumoniae* | 1 | >16 | 1 | >32 | 0.25 | <0.12 | <1 | 1 | (<0.25) | KPC-3; TEM-OSBL |
| Italy | *K. pneumoniae* | 1 | >16 | 1 | >32 | 0.25 | <0.12 | <1 | 1 | (<0.25) | KPC-3; SHV-OSBL; TEM-OSBL |
| Italy | *K. pneumoniae* | 2 | >16 | 8 | >32 | 0.5 | 0.5 | <1 | 1 | (<0.25) | KPC-3; SHV-OSBL |
| Italy | *K. pneumoniae* | 0.25 | >16 | 8 | >32 | 0.25 | 0.5 | <1 | 0.5 | (<0.25) | KPC-3; SHV-OSBL; TEM-OSBL |
| Italy | *K. pneumoniae* | 2 | >16 | 4 | >32 | 1 | 0.5 | <1 | 2 | (<0.25) | KPC-3; SHV-OSBL |
| Italy | *K. pneumoniae* | 0.5 | >16 | 2 | >32 | 0.5 | 0.25 | <1 | 1 | (<0.25) | KPC-3; SHV-OSBL |
| Italy^b^ | *K. pneumoniae* | 2 | >16 | 2 | >32 | 16 | 4 | <1 | 2 | (<0.25) | CTX-M-15; KPC-3; OXA-48; SHV-1 |
| Italy | *Klebsiella* sp. | 0.5 | >16 | 0.5 | >32 | <0.06 | 0.25 | <1 | <0.25 | (<0.25) | CTX-M-1-240G; KPC-3; SHV-OSBL; TEM-OSBL |
| Italy | *K. pneumoniae* | 0.5 | >16 | 1 | >32 | 1 | 0.5 | <1 | 1 | (<0.25) | CTX-M-1-24G; KPC-3; SHV-OSBL; TEM-OSBL |
| Italy | *K. pneumoniae* | 2 | 16 | 4 | >32 | <0.06 | 0.25 | <1 | 0.5 | (<0.25) | CTX-M-1-24G; KPC-3; SHV-OSBL; TEM-OSBL |
| Italy | *K. pneumoniae* | 2 | >16 | 1 | >32 | 0.5 | 0.5 | <1 | 1 | (<0.25) | KPC-3; SHV-OSBL |
| Italy | *K. pneumoniae* | 1 | >16 | 4 | >32 | 2 | 2 | <1 | 2 | (<0.25) | KPC-3; SHV-OSBL |
| Italy | *K. pneumoniae* | 1 | >16 | 4 | >32 | 1 | 0.5 | <1 | 2 | (<0.25) | KPC-3; SHV-OSBL |
| Italy | *K. pneumoniae* | 1 | >16 | 2 | >32 | 4 | 0.5 | <1 | 4 | (<0.25) | CTX-M-1-24G; KPC-3; SHV-OSBL; TEM-OSBL |
| Italy^a^ | *K. pneumoniae* | 4 | >16 | 2 | >32 | 1 | 0.5 | <1 | 2 | (<0.25) | KPC-3; OXA-9-Trunc; SHV-11; TEM-1 |
| Italy | *K. pneumoniae* | 2 | >16 | 1 | >32 | 0.5 | <0.12 | <1 | 2 | (>8) | KPC-2; SHV-OSBL; TEM-OSBL |
| Italy | *K. pneumoniae* | 0.5 | >16 | >16 | >32 | >32 | 4 | 2 | 2 | (<0.25) | KPC-3; SHV-OSBL; TEM-OSBL |
| Italy | *K. pneumoniae* | 2 | >16 | <0.25 | >32 | 0.5 | <0.12 | <1 | 1 | (>8) | CMY-2-TYPE; KPC-3; SHV-OSBL; TEM-OSBL |
| Italy | *K. pneumoniae* | 1 | >16 | 1 | >32 | 0.5 | 0.25 | <1 | 1 | (>8) | KPC-3; SHV-OSBL; TEM-OSBL |
| Italy | *Enterobacter* sp. | 2 | >16 | >16 | >32 | 32 | 16 | <1 | 1 | (<0.25) | VIM-1 |
| Italy^a^ | *E. cloacae* | 8 | >16 | >16 | >32 | 32 | 16 | <1 | 8 | (<0.25) | ACT-24; NDM-1 |
| Italy | *C. braakii* | 2 | >16 | >16 | >32 | >32 | >16 | <1 | 16 | (<0.25) | KPC-3; SHV-OSBL; VIM-1 |
| Italy | *Enterobacter* sp. | 2 | >16 | >16 | >32 | 16 | 8 | <1 | 1 | (1) | DHA-TYPE; VIM-1 |
| Italy | *K. pneumoniae* | 0.5 | 16 | 0.5 | 32 | <0.06 | <0.12 | <1 | <0.25 | (<0.25) | KPC-3; SHV-OSBL |
| Italy | *K. pneumoniae* | 1 | >16 | 8 | >32 | 2 | 2 | <1 | 2 | (<0.25) | KPC-3; SHV-OSBL |
| Italy | *K. pneumoniae* | 2 | >16 | 2 | >32 | 2 | 1 | <1 | 1 | (<0.25) | KPC-3; SHV-OSBL |
| Italy | *K. pneumoniae* | 1 | >16 | 8 | >32 | 4 | 0.5 | <1 | 2 | (<0.25) | KPC-3; SHV-OSBL |
| Italy | *K. pneumoniae* | 1 | >16 | 4 | >32 | 2 | 0.5 | <1 | 4 | (<0.25) | KPC-3; SHV-OSBL |
| Italy | *K. pneumoniae* | 2 | >16 | 2 | >32 | 2 | 1 | <1 | 1 | (<0.25) | KPC-3; SHV-OSBL |
| Italy | *K. pneumoniae* | 1 | >16 | 4 | >32 | 2 | 1 | <1 | 1 | (<0.25) | KPC-3; SHV-OSBL |
| Italy | *K. pneumoniae* | 1 | >16 | 2 | >32 | 1 | 0.5 | <1 | 2 | (<0.25) | KPC-3; SHV-OSBL |
| Italy | *K. pneumoniae* | 1 | >16 | 4 | >32 | 4 | 4 | <1 | 2 | (<0.25) | KPC-3; SHV-OSBL |
| Italy | *K. pneumoniae* | 2 | >16 | 2 | >32 | 0.5 | 0.5 | <1 | 1 | (<0.25) | KPC-3; SHV-OSBL |
| Italy | *K. pneumoniae* | 1 | >16 | 4 | >32 | 2 | 0.5 | <1 | 2 | (<0.25) | KPC-3; SHV-OSBL |
| Italy^b^ | *E. coli* | 8 | >16 | >16 | >32 | <0.06 | 0.5 | <1 | <0.25 | (<0.25) | CTX-M-15; KPC-31; OXA-1; SHV-28; TEM-1 |
| Italy | *K. pneumoniae* | 1 | >16 | 4 | >32 | 0.5 | 0.5 | <1 | 1 | (<0.25) | KPC-3; SHV-OSBL |
| Italy | *K. pneumoniae* | 2 | >16 | 1 | >32 | 0.5 | 0.25 | <1 | 1 | (<0.25) | KPC-3; SHV-OSBL |
| Italy | *K. pneumoniae* | 2 | >16 | >16 | >32 | >32 | >16 | <1 | 2 | (<0.25) | CMY-2-TYPE; CTX-M-1-24G; NDM-1; SHV-OSBL |
| Italy | *K. pneumoniae* | 2 | >16 | >16 | >32 | >32 | >16 | <1 | 4 | (<0.25) | CMY-2-TYPE; CTX-M-1-24G; NDM-1; SHV-OSBL |
| Italy | *E. coli* | 1 | 16 | >16 | >32 | 32 | 16 | <1 | 0.5 | (<0.25) | CMY-2-TYPE; NDM-1 |
| Italy | *K. pneumoniae* | 2 | >16 | <0.25 | >32 | 1 | 0.5 | <1 | 1 | (<0.25) | CTX-M-1-TYPE; KPC-3; SHV-OSBL; TEM-OSBL |
| Italy | *K. pneumoniae* | 2 | >16 | 1 | >32 | 1 | 0.25 | <1 | 2 | (<0.25) | KPC-3; SHV-OSBL |
| Italy | *K. pneumoniae* | 2 | >16 | 1 | >32 | 1 | 0.5 | <1 | 1 | (<0.25) | KPC-3; SHV-OSBL |
| Italy | *K. pneumoniae* | 2 | >16 | 2 | >32 | 1 | 0.5 | <1 | 2 | (<0.25) | KPC-3; SHV-OSBL |
| Italy^b^ | *K. pneumoniae* | 4 | >16 | 8 | >32 | 1 | 0.25 | <1 | 2 | (<0.25) | KPC-3; SHV-1 |
| Italy | *K. pneumoniae* | 0.5 | >16 | 1 | >32 | <0.06 | 0.25 | <1 | <0.25 | (<0.25) | CTX-M-1-240G; KPC-3; TEM-OSBL |
| Italy | *K. pneumoniae* | 1 | >16 | 4 | >32 | 1 | 0.5 | <1 | 2 | (<0.25) | KPC-2; SHV-OSBL |
| Italy | *K. pneumoniae* | 1 | >16 | 2 | >32 | 0.25 | 0.5 | <1 | 0.5 | (<0.25) | CTX-M-1-24G; KPC-3; SHV-OSBL; TEM-OSBL |
| Italy | *K. pneumoniae* | 1 | >16 | 2 | >32 | <0.06 | 0.25 | <1 | <0.25 | (<0.25) | KPC-3; SHV-OSBL; TEM-OSBL |
| Italy | *K. pneumoniae* | 2 | >16 | 2 | >32 | 1 | 1 | <1 | 2 | (<0.25) | KPC-3; SHV-OSBL; TEM-OSBL |
| Italy | *K. pneumoniae* | 2 | >16 | 2 | >32 | 0.5 | 0.5 | <1 | 2 | (<0.25) | KPC-3; TEM-OSBL |
| Italy | *K. pneumoniae* | 0.12 | 16 | 1 | >32 | <0.06 | 1 | <1 | <0.25 | (0.5) | KPC-3; SHV-OSBL; TEM-OSBL |
| Italy | *K. pneumoniae* | 2 | >16 | 2 | >32 | 1 | 1 | <1 | 1 | (<0.25) | KPC-3; SHV-OSBL; TEM-OSBL |
| Italy | *K. pneumoniae* | 1 | >16 | 2 | >32 | <0.06 | 0.25 | <1 | <0.25 | (<0.25) | KPC-3; SHV-OSBL; TEM-OSBL |
| Italy | *K. pneumoniae* | 2 | >16 | 2 | >32 | 0.5 | 0.25 | <1 | 0.5 | (<0.25) | CTX-M-1-24G; KPC-3; SHV-OSBL; TEM-OSBL |
| Italy | *K. pneumoniae* | 2 | >16 | 2 | >32 | 1 | 0.5 | <1 | 2 | (<0.25) | KPC-3; SHV-OSBL; TEM-OSBL |
| Italy | *K. pneumoniae* | 1 | >16 | 2 | >32 | <0.06 | 0.25 | <1 | 0.5 | (<0.25) | KPC-3; SHV-OSBL; TEM-OSBL |
| Italy | *K. pneumoniae* | 0.5 | >16 | 8 | >32 | 16 | 4 | <1 | 4 | (<0.25) | KPC-3; SHV-OSBL |
| Italy | *E. cloacae* | 2 | 16 | >16 | >32 | 16 | 16 | <1 | 0.5 | (<0.25) | SHV-ESBL; VIM-1; TEM-OSBL |
| Italy | *K. pneumoniae* | 2 | 16 | 1 | >32 | <0.06 | <0.12 | <1 | <0.25 | (<0.25) | KPC-3; SHV-OSBL; TEM-OSBL |
| Italy | *K. pneumoniae* | 1 | >16 | >16 | >32 | >32 | >16 | <1 | 4 | (<0.25) | CTX-M-1-24G; NDM-1; SHV-OSBL; TEM-OSBL |
| Italy | *K. pneumoniae* | 0.25 | >16 | 1 | >32 | 0.5 | 0.25 | <1 | 1 | (<0.25) | KPC-3; SHV-OSBL; TEM-OSBL |
| Italy | *K. pneumoniae* | 0.5 | >16 | <0.25 | >32 | <0.06 | 0.25 | <1 | 0.5 | (<0.25) | CTX-M-1-240G; KPC-2; SHV-OSBL; TEM-OSBL |
| Italy | *K. pneumoniae* | 0.5 | >16 | 0.5 | >32 | <0.06 | 0.25 | <1 | 0.5 | (4) | CTX-M-1-240G; KPC-2; SHV-OSBL; TEM-OSBL |
| Italy | *K. pneumoniae* | 0.5 | 16 | 0.5 | >32 | <0.06 | 0.25 | <1 | <0.25 | (<0.25) | CTX-M-1-240G; KPC-2; SHV-OSBL; TEM-OSBL |
| Spain | *K. pneumoniae* | 1 | 16 | 1 | >32 | <0.06 | <0.12 | <1 | <0.25 | (<0.25) | KPC-3; SHV-OSBL; TEM-OSBL |
| Spain^b^ | *K. pneumoniae* | 2 | >16 | 2 | >32 | 0.5 | 0.25 | <1 | 2 | (<0.25) | KPC-3; OXA-9-trunc; SHV-11; TEM-Multicopy |
| Spain | *K. pneumoniae* | 2 | >16 | 1 | >32 | 1 | 0.5 | <1 | 2 | (<0.25) | KPC-3; SHV-OSBL; TEM-OSBL |
| Spain | *K. pneumoniae* | 2 | >16 | 2 | >32 | 0.5 | 0.25 | <1 | 1 | (<0.25) | KPC-3; SHV-OSBL; TEM-OSBL |
| Spain | *K. pneumoniae* | 2 | >16 | 8 | >32 | 8 | 4 | <1 | 4 | (<0.25) | KPC-3; SHV-OSBL; TEM-OSBL |
| Spain | *K. pneumoniae* | 2 | >16 | 2 | >32 | 0.5 | 2 | <1 | 2 | (<0.25) | KPC-3; SHV-OSBL; TEM-OSBL |
| Spain | *K. pneumoniae* | 2 | >16 | >16 | >32 | 8 | 8 | <1 | 1 | (<0.25) | CTX-M-9-24D; SHV-OSBL; VIM-1 |
| Spain^a^ | *E. cloacae* | 32 | >16 | >16 | >32 | 32 | 16 | <1 | 0.5 | (<0.25) | CMH-Type; CTX-M-9; OXA-1-trunc; SHV-12; VIM-1 |
| Spain | *K. pneumoniae* | 0.5 | 16 | 1 | >32 | 16 | 8 | <1 | 4 | (<0.25) | CTX-M-1-24G; OXA-48; SHV-OSBL; TEM-OSBL |
| Spain | *K. pneumoniae* | 1 | >16 | 1 | >32 | 16 | 4 | 32 | 2 | (0.5) | CTX-M-1-24G; OXA-48; SHV-OSBL; TEM-OSBL |
| Spain^b^ | *K. oxytoca* | 0.5 | >16 | >16 | >32 | 32 | >16 | <1 | 4 | (<0.25) | DHA-TYPE; NDM-1 |
| Spain^a^ | *K. oxytoca* | 4 | >16 | >16 | >32 | >32 | >16 | <1 | 2 | (<0.25) | NDM-1; OXA-101; OXY-1-3; TEM-250; TEM-OSBL |
| Spain^a^ | *E. bugandensis* | 16 | >16 | >16 | >32 | 32 | >16 | <1 | 8 | (0.5) | ACT-52; NDM-1; OXA-1 |
| Spain | *E. bugandensis* | 2 | 16 | 0.5 | 8 | 16 | 8 | <1 | 0.5 | (<0.25) | MIR-TYPE; OXA-48 |
| Spain^a^ | *E. cloacae* complex | 8 | >16 | >16 | >32 | >32 | >16 | <1 | 16 | (>8) | ACT-52; NDM-1; OXA-1 |
| Spain^a^ | *E. bugandensis* | 4 | >16 | >16 | >32 | >32 | >16 | <1 | 16 | (>8) | ACT-52; NDM-1 |
| Spain | *E. cloacae* complex | 1 | >16 | 1 | 32 | 0.5 | 2 | <1 | 2 | (>8) | KPC-1 |
| Spain^b^ | *C. koseri* | 8 | >16 | >16 | >32 | 32 | >16 | <1 | 1 | (<0.25) | CKO-New variant; NDM-1 |
| Spain^a^ | *E. bugandensis* | 16 | >16 | >16 | >32 | >32 | >16 | <1 | 16 | (0.5) | ACT-52; NDM-1; OXA-1 |
| Spain^a^ | *E. bugandensis* | 4 | >16 | >16 | >32 | >32 | >16 | <1 | 4 | (>8) | ACT-52; NDM-1 |
| UK | *K. pneumoniae* | 0.06 | >16 | 0.5 | >32 | <0.06 | 0.25 | <1 | <0.25 | (<0.25) | KPC-2; SHV-OSBL; TEM-OSBL |
| UK | *K. pneumoniae* | 2 | >16 | >16 | >32 | 32 | 16 | <1 | 1 | (<0.25) | CTX-M-1-24G; KPC-TYPE; NDM-1; SHV-OSBL; TEM-OSBL |
| UK | *K. oxytoca* | 2 | >16 | >16 | >32 | 16 | 8 | <1 | 0.5 | (<0.25) | VIM-1 |
| UK^a^ | *E. cloacae* | 4 | >16 | >16 | >32 | 32 | 16 | <1 | 4 | (<0.25) | ACT-24; ACT-5-TYPE; NDM-1; TEM-1; TEM-OSBL |
| UK | *K. pneumoniae* | 0.25 | >16 | 1 | >32 | <0.06 | 0.25 | <1 | <0.25 | (<0.25) | KPC-2; SHV-ESBL |

β-Lactamase data were generated from PCR unless otherwise indicated. Colistin data are shown in parentheses as colistin is not recommended for monotherapy and is not associated with a clinical monotherapy breakpoint (as per EUCAST v.14.0 guidance).^3^

^a^Isolates with β-lactamase gene data collated from PCR and WGS.

^b^Cefiderocol-resistant isolates with β-lactamase gene data from WGS.

ATM-AVI, aztreonam-avibactam; CST, colistin; C‑T, ceftolozane‑tazobactam; CZA, ceftazidime-avibactam; ESBL, extended-spectrum β lactamases; FDC, cefiderocol; FEP-TAN, cefepime-taniborbactam; KPC, *Klebsiella pneumoniae* carbapenemase; I-R, imipenem-relebactam; MBL, metallo-β-lactamase; MEM, meropenem; MVB, meropenem-vaborbactam; NDM, New Delhi MBL; OSBL, original-spectrum β-lactamase; Trunc, truncated; UK, United Kingdom; VIM, Verona integron-borne MBL; WGS, whole genome sequencing.

**TABLE S8** Participating sites

| **Country** | **Centre** | **Lead investigator** |
| --- | --- | --- |
| Austria | AKH Wien | Birgit Willinger |
| France | CH de la Côte Basque | David Leyssene |
| France | CH de Valenciennes | Christian Cattoen |
| France | CHRU de Nancy - Hôpital Brabois Adultes | Corentine Alauzet |
| France | CHRU de Strasbourg | Pierre Boyer |
| France | CHU de Bordeaux | Véronique Dubois |
| France | CHU de Besançon - Hôpital Jean-Minjoz | Katy Jeannot |
| France | CHU de Nantes | Stephane Corvec |
| France | CHU de Nîmes | Jean-Philippe Lavigne |
| France | CHU de Reims | Thomas Guillard |
| France | HIA Bégin | Audrey Merens Gontier |
| France | Hôpital Bicêtre | Thierry Naas |
| Germany | Universitätsklinikum Hamburg-Eppendorf | Holger Rohde |
| Germany | Medizinische Hochschule Hannover | Stefan Ziesing |
| Germany | Justus-Liebig-Universität Giessen | Can Imirzalioglu |
| Germany | Krankenhaus Nordwest Zentralinstitut für Laboratoriumsmedizin | Klaus-Peter Hunfeld |
| Germany | LMU München | Jette Jung |
| Germany | Ruhr-Universität Bochum | Sören Gatermann |
| Germany | Universitatsklinikum Jena | Mathias Pletz |
| Italy | AOU Città della Salute e della Scienza di Torino | Gabriele Bianco |
| Italy | AOU Policlinico Paolo Giaccone | Anna Giammanco |
| Italy | ASST Monza - Ospedale di Desio | Davide Carcione |
| Italy | Azienda Policlinico Umberto I di Roma | Giammarco Raponi |
| Italy | Fondazione IRCCS Ca’ Granda Ospedale Maggiore Policlinico | Caterina Matinato |
| Italy | Istituti Fisioterapici Ospitalieri | Enea Gino Di Domenico |
| Italy | IRCCS Policlinico di Sant’Orsola-Malpighi | Paolo Gaibani |
| Italy | IRCCS Ospedale Policlinico San Martino | Anna Marchese |
| Italy | Università degli studi di Foggia | Fabio Arena |
| Italy | Università degli Studi di Firenze | Claudia Niccolai |
| Italy | Università degli Studi di Catania | Stefania Stefani |
| Spain | Hospital Clínic de Barcelona | Cristina Pitart |
| Spain | Hospital Universitario Cruces | Jose Luis Barrios |
| Spain | Hospital General Universitario Gregorio Marañón | Emilia Cercenado |
| Spain | Hospital Universitario de A Coruña | German Bou |
| Spain | Hospital Nuestra Señora del Prado | Alicia Beteta Lopez |
| Spain | Hospital Universitario Ramón y Cajal | Rafael Canton |
| Spain | Hospital Universitario y Politécnico La Fe | Jose Lopez Hontangas |
| Spain | Hospital Universitario Reina Sofía | Irene Gracia-Ahufinger |
| Spain | Hospital Universitario Son Espases | Antonio Oliver |
| Spain | Hospital Universitario Virgen Macarena | Lorena Lopez-Cerero |
| Spain | Hospital Vall d’Hebron | Nieves Larrosa |
| United Kingdom | Queen Mary University of London | David Wareham |
| United Kingdom | Freeman Hospital, Newcastle Hospitals NHS Foundation Trust | John Perry |
| United Kingdom | Queen Elizabeth Hospital Birmingham, University Hospitals Birmingham NHS Foundation Trust | Anna Casey |
| United Kingdom | Leeds Teaching Hospitals NHS Trust | Jasvir Nahl |
| United Kingdom | Manchester University Hospital, NHS Foundation Trust | Daniel Hughes |
| United Kingdom | Glasgow Royal Infirmary, NHS Greater Glasgow and Clyde | Michael Coyne |
| United Kingdom | Queen’s Medical Centre, Nottingham University Hospitals NHS Trust | Michelle Lister |
| United Kingdom | Southmead Hospital, North Bristol NHS Trust | Marie Attwood |

AKH Wein, Allgemeines Krankenhaus der Stadt Wien; AOU, Azienda Ospedaliera Universitaria; CH, Centre Hospitalier; CHU, CH Universitaire; CHRU, CH Régional Universitaire; HIA, L'hôpital d'instruction des armées; IRCCS, Istituto di Ricovero e Cura a Carattere Scientifico; LMU, Ludwig-‍Maximilians-‍Universität; NHS, National Health Service.

**TABLE S9** Suppliers of antimicrobial agents

| **Agent** | **Supplier** | **Product code** |
| --- | --- | --- |
| Avibactam sodium | BioChemPartner (China) | BCP08818 |
| Aztreonam | USP (North Bethesda, MA, USA) | 1046205 |
| Cefepime hydrochloride | Sigma-Aldrich (St. Louis, MO, USA) | PHR1763 |
| Cefiderocol | AVEP (IHMA 3825) | N/A |
| Ceftolozane sulfate | Adooq Bioscience (Irvine, CA, USA) | A13801 |
| Colistin sulfate | USP | 1148001 |
| Imipenem | USP | 1337809 |
| Meropenem | USP | 1392454 |
| Relebactam | MedChemExpress (Monmouth Junction, NJ, USA) | HY-16752 |
| Taniborbactam | MedChemExpress | 2244235-49-0 |
| Tazobactam | Selleckchem (Houston, TX, USA) | S3077 |
| Vaborbactam | MedChemExpress | 1360457-460 |

Excludes avibactam and ceftazidime, as Sensititre™ freeze dried panels (Thermo Fisher Scientific Inc., Waltham, MA, USA) were used for ceftazidime‑avibactam testing.

AVEP, Antimicrobial Voluntary Evaluation Program; IHMA, International Health Management Associates; N/A, not applicable; USA, United States of America; USP, United States pharmacopeia.

**TABLE S10** Polymerase chain reaction primer sequences used for detection of β-lactamase genes in meropenem-resistant, cefiderocol‑susceptible Enterobacterales isolates

| **Target gene** | **Primer name** | **Primer sequence 5'-3'** | **Annealing temperature, °C** |
| --- | --- | --- | --- |
| TEM | TEM-3 | CATTTCCGTGTCGCCCTTATTC | 59 |
|  | TEM-4 | CGTTCATCCATAGTTGCCTGAC |  |
| SHV | SHV-5 | CCTTTAAAGTAGTGCTCTGC | 59 |
|  | SHV-6 | TTCGCTGACCGGCGAGTAGT |  |
| VEB | VEB-F | CATTTCCCGATGCAAAGCGT | 59 |
|  | VEB-R | CGAAGTTTCTTTGGACTCTG |  |
| PER | PERpan-F | TAGGYGTTGCMGTRTGGGG | 59 |
|  | PERpan-R | GGTTTCRACCATCCAYTTCC |  |
| GES | GES-F | AGTCGGCTAGACCGGAAAG | 59 |
|  | GES-R | TTTGTCCGTGCTCAGGAT |  |
| OXA-48 group | OXA-48-F | GCTTGATCGCCCTCGATT | 59 |
|  | OXA-48-R2 | GATTTGCTSSGTRGCCGAAA |  |
| 16S | 16s-519r | GWATTACCGCGGCKGCTG | 59 |
|  | U341F | CCTACGGGRSGCAGCAG |  |
| IMP | IMP2-F | GGAATAGAGTGGCTTAAYTCTC | 59 |
|  | IMP2r4 | GGTTTAAYAAARCAACCRCC |  |
| VIM | VIM-F | GATGGTGTTTGGTCGCATA | 59 |
|  | VIM-R3 | CGAATGCGCAGCACCAGGA |  |
| SPM | SPM-F | AAAATCTGGGTACGCAAACG | 59 |
|  | SPM-R | ACATTATCCGCTGGAACAGG |  |
| NDM | NDM-F | CCGTATGAGTGATTGCGGCG | 59 |
|  | NDM-R | GCCCAATATTATGCACCCGG |  |
| KPC | KPCy-F | TGTCACTGTATCGCCGTC | 59 |
|  | KPCy-R | CTCAGTGCTCTACAGAAAACC |  |
| GIM | GIM-F | TCGACACACCTTGGTCTGAA | 59 |
|  | GIM-R | AACTTCCAACTTTGCCATGC |  |
| MOX | MOX-MF | GCTGCTCAAGGAGCACAGGAT | 65 |
|  | MOX-MR | CACATTGACATAGGTGTGGTGC |  |
| FOX | FOX-MF | AACATGGGGTATCAGGGAGATG | 65 |
|  | FOX-MR | CAAAGCGCGTAACCGGATTGG |  |
| ACC | ACC-MF | AACAGCCTCAGCAGCCGGTTA | 65 |
|  | ACC-MR | TTCGCCGCAATCATCCCTAGC |  |
| CMY II | CIT-MF | TGGCCAGAACTGACAGGCAAA | 65 |
|  | CIT-MR | TTTCTCCTGAACGTGGCTGGC |  |
| ACT/MIR | EBC-MF | TCGGTAAAGCCGATGTTGCGG | 65 |
|  | EBC-MR | CTTCCACTGCGGCTGCCAGTT |  |
| DHA | DHA-MF | AACTTTCACAGGTGTGCTGGGT | 65 |
|  | DHA-MR | CCGTACGCATACTGGCTTTGC |  |
| CTX-M-1 | CTX-M1f2 | AAAAATCACTGCGCCAGTTC | 58 |
|  | CTX-M1r2 | AGCTTATTCATCGCCACGTT |  |
| CTX-M-2 | CTX-M2f2 | CGACGCTACCCCTGCTATT | 58 |
|  | CTX-M2r2 | CCAGCGTCAGATTTTTCAGG |  |
| CTX-M-9 | CTX-M9f2 | CAAAGAGAGTGCAACGGATG | 58 |
|  | CTX-M9r2 | ATTGGAAAGCGTTCATCACC |  |
| CTX-M-8 | CTX-M-8A | TCGCGTTAAGCGGATGATGC | 58 |
|  | CTX-M-8-25B | AACCCACGATGTGGGTAGC |  |
| CTX-M-25 | CTX-M-25A | GCACGATGACATTCGGG | 58 |
|  | CTX-M-8-25B | AACCCACGATGTGGGTAGC |  |

ACC, Ambler Class C; F, forward primer; GES, Guiana extended-spectrum β-lactamase; GIM, German imipenemase; IMP, imipenemase; KPC, *Klebsiella ‍pneumoniae* carbapenemase; MBL, metallo-β-lactamase; MF, modified F; MR, modified R; NDM, New Delhi MBL; OXA, oxacillinase; PER, *Pseudomonas* extended resistant; R, reverse primer; VEB, Vietnamese extended-spectrum β-lactamase; VIM, Verona integron-borne MBL.

**TABLE S11** Species-specific reference genes used for whole genome sequencing of cefiderocol-resistant Enterobacterales isolates

| **Species** | **Strain** | **Gene name** | **Nucleotide accession** | **Locus tag** | **Amino acid accession** |
| --- | --- | --- | --- | --- | --- |
| *Citrobacter amalonaticus* | FDAARGOS_165 | *ftsI* | NZ_CP014070 | AL524_RS48185 | WP_042997941.1 |
|  |  | *ompC* |  | AL524_RS36695 | WP_043000069.1 |
|  |  | *ompF* |  | AL524_RS43990 | WP_104010347.1 |
|  |  | *exbB* |  | AL524_RS49235 | WP_042998151.1 |
|  |  | *exbD* |  | AL524_RS49230 | WP_042324207.1 |
|  |  | *tonB* |  | AL524_RS39930 | WP_054176350.1 |
| *Citrobacter koseri* | ATCC BAA-895 | *ftsI* | NZ_009792 | CKO_RS14080 | WP_012134078.1 |
|  |  | *ompC* |  | CKO_RS02455 | WP_012131537.1 |
|  |  | *ompF* |  | CKO_RS09060 | WP_012132994.1 |
|  |  | *exbB* |  | CKO_RS18775 | WP_024130953.1 |
|  |  | *exbD* |  | CKO_RS18770 | WP_012135139.1 |
|  |  | *tonB* |  | CKO_RS05720 | WP_012132209.1 |
| *Enterobacter cloacae* | ATCC 13047 | *ftsI* | NC_014121 | ECL_00881 | YP_003611393.1 |
|  |  | *ompC* |  | ECL_03519 | YP_003614002.1 |
|  |  | *ompF* |  | ECL_02724 | YP_003613214.1 |
|  |  | *cirA*-like |  | ECL_03465 | YP_003613948.1 |
|  |  | *fiu*-like |  | ECL_00954 | YP_003611466.1 |
|  |  | *exbB* |  | ECL_04329 | YP_003614808.1 |
|  |  | *exbD* |  | ECL_04328 | YP_003614807.1 |
|  |  | *tonB* |  | ECL_01647 | YP_003612154.1 |
| *Enterobacter hormaechei* | RHBSTW-00086 | *tonB* | NZ_CP058157 | HV061_RS10095 | WP_063199392.1 |
| *Enterobacter kobei* | UCI 24 | *fiu*-like | NZ_KI973153 | P827_RS06160 | WP_014882612.1 |
|  |  | *tonB* |  | P827_RS14395 | WP_014884119.1 |
| *Enterobacter ludwigii* | EN-119 | *fiu*-like | NZ_CP017279 | BH714_RS18640 | WP_040018652.1 |
|  |  | *tonB* |  | BH714_RS08715 | WP_025203933.1 |
| *Escherichia coli* | K12 MG1655 | *ftsI* | NC_000913 | b0084 | NP_414626.1 |
|  |  | *ompC* |  | b2215 | NP_416719.1 |
|  |  | *ompF* |  | b0929 | NP_415449.1 |
|  |  | *cirA*-like |  | b2155 | NP_416660.1 |
|  |  | *fiu*-like |  | b0805 | NP_415326.1 |
|  |  | *exbB* |  | b3006 | NP_417479.1 |
|  |  | *exbD* |  | b3005 | NP_417478.1 |
|  |  | *tonB* |  | b1252 | NP_415768.1 |
| *Klebsiella aerogenes* | ATCC 13048 | *ftsI* | NZ_QVM01000^a^ | D0N43_RS13175 | WP_015368251.1 |
|  |  | *ompC* |  | D0N43_RS09530 | WP_015706141.1 |
|  |  | *ompF* |  | D0N43_RS03375 | WP_015368078.1 |
|  |  | *cirA*-like |  | D0N43_RS09290 | WP_015706114.1 |
|  |  | *exbB* |  | D0N43_RS06605 | WP_015369670.1 |
|  |  | *exbD* |  | D0N43_RS06600 | WP_015369671.1 |
|  |  | *tonB* |  | D0N43_RS12330 | WP_015705108.1 |
| *Klebsiella oxytoca* | KONIH1 | *ftsI* | NZ_CP008788 | KONIH1_RS04535 | WP_004098582.1 |
|  |  | *ompC* |  | KONIH1_RS19805 | WP_014230214.1 |
|  |  | *ompF* |  | KONIH1_RS09985 | WP_042944813.1 |
|  |  | *cirA*-like |  | KONIH1_RS19550 | WP_032693692.1 |
|  |  | *fiu*-like |  | KONIH1_RS08940 | WP_032694520.1 |
|  |  | *exbB* |  | KONIH1_RS06930 | WP_004137930.1 |
|  |  | *exbD* |  | KONIH1_RS24615 | WP_014226862.1 |
| *Klebsiella pneumoniae* | ATCC 13883 | *ftsI* | NZ_KN046818 | DR88_RS22130 | WP_002888559.1 |
|  |  | *ompC* (*ompK36*) |  | DR88_RS05545 | WP_004149145.1 |
|  |  | *ompF* (*ompK35*) |  | DR88_RS17700 | WP_004141771.1 |
|  |  | *cirA*-like |  | DR88_RS05330 | WP_032443975.1 |
|  |  | *fiu*-like |  | DR88_RS16150 | WP_023316750.1 |
|  |  | *exbB* |  | DR88_RS09680 | WP_004174395.1 |
|  |  | *exbD* |  | DR88_RS09675 | WP_002916785.1 |
|  |  | *tonB* |  | DR88_RS12390 | WP_025861279.1 |
| *Klebsiella variicola* | DSM 15968 | *ftsI* | CP010523 | SP68_18905 | AJE91227.1 |
|  |  | *ompC* |  | SP68_07235 | AJE89055.1 |
|  |  | *ompF* |  | SP68_23345 | AJE92057.1 |
|  |  | *cirA*-like |  | SP68_06925 | AJE88998.1 |
|  |  | *fiu*-like |  | SP68_25270 | AJE92416.1 |
|  |  | *exbB* |  | SP68_11195 | AJE89801.1 |
|  |  | *exbD* |  | SP68_11190 | AJE89800.1 |
|  |  | *tonB* |  | SP68_04560 | AJE88564.1 |
| *Serratia marcescens* | UMH2 | *ftsI* | NZ_CP018924 | BVG88_RS00340 | WP_004932790.1 |
|  |  | *ompC* |  | BVG88_RS14160 | WP_033643836.1 |
|  |  | *ompF* |  | BVG88_RS05490 | N/A^a^ |
|  |  | *exbB* |  | BVG88_RS18730 | WP_080273417.1 |
|  |  | *exbD* |  | BVG88_RS18725 | WP_004937307.1 |
|  |  | *tonB* |  | BVG88_RS10685 | WP_033643281.1 |

References for several siderophore uptake genes in *C. koseri*, *K. oxytoca* or *S. marcescens* were not available and are excluded so there is no reference gene or data for these.

^a^Reference sequence is truncated through a nonsense mutation; the full-length protein is considered to be 373 amino acids.

N/A, not applicable.

**SUPPLEMENTARY REFERENCES**

1. European Committee on Antimicrobial Susceptibility Testing. 2024. Breakpoint tables for interpretation of MICs and zone diameters, Version 14.0. https://www.eucast.org/fileadmin/src/media/PDFs/EUCAST_files/Breakpoint_tables/v_14.0_Breakpoint_Tables.pdf. Accessed April 2024.
2. Clinical and Laboratory Standards Institute. 2023. M100: Performance Standards for Antimicrobial Susceptibility Testing. 33rd Edition. CLSI, https://clsi.org/standards/products/microbiology/documents/m100/. Accessed April 2024.
3. Breakpoint Committee consultation on Colistin. EUCAST, European Committee on Antimicrobial Susceptibility Testing. Colistin Breakpoints - Guidance document 2022.
